# Decoding Biomolecular Phase Separation: From Physiological Roles to Pathological Mechanisms and Therapeutic Biomaterials Design

**DOI:** 10.1002/advs.78087

**Published:** 2026-10-08

**Authors:** Jiawei Zhu, Jingbo Dong, Qiufang Gong, Xuejiao Song, Zhifa Shen, Chao Liang

**Affiliations:** ^1^ College of Laboratory Medicine and Life sciences Cixi Institute of Cancer Research Cixi People's Hospital Wenzhou Medical University Ningbo Zhejiang China; ^2^ Cixi Biomedical Research Institute Wenzhou Medical University Ningbo Zhejiang China; ^3^ School of Physical and Mathematical Sciences Nanjing Tech University (NanjingTech) Nanjing China

**Keywords:** biomolecular phase separation, BPS‐inspired biomaterials, membraneless organelles, pathological phase transition, phase separation‐targeted therapy

## Abstract

Biomolecular phase separation (BPS), encompassing liquid‐liquid, liquid‐gel, and liquid‐solid transitions, has emerged as a fundamental principle governing cellular organization and function. Dysregulation of these phase states acts as a pathogenic driver of diverse intractable diseases, including neurodegeneration, cancer, and autoimmune disorders. The intrinsic properties of biomolecular condensates, self‐assembly, dynamic reversibility, and environmental responsiveness, also offer a robust blueprint for next‐generation biomaterial design. Despite rapid progress across the field, a cohesive framework linking phase transition physics, pathological mechanisms, and translational engineering remains absent. Here, we delineate the full continuum of liquid‐liquid, liquid‐gel and liquid‐solid phase separation, elucidating how dynamic interconversion across phase states regulates core physiological processes and drives the pathogenesis of neurodegeneration, cancer and autoimmune disorders. Meanwhile, we establish a property‐design‐function paradigm that translates the physicochemical rules of phase separation into rational biomaterial engineering. Building on this mechanistic foundation, we systematically evaluate emerging therapeutic strategies that target aberrant condensates via either BPS induction or inhibition. We also highlight BPS‐inspired biomaterials across cell‐mimetic systems, responsive drug delivery platforms, bioactive scaffolds, and high‐performance imaging probes. Key translational challenges and forward‐looking pathways are discussed alongside these advances. This review provides an integrated cross‐disciplinary perspective bridging physicochemical fundamentals, pathological implications, and biomedical engineering.

## Graphical abstract

This review systematically elaborates on the mechanisms underlying physiological regulation and pathological dysregulation of biomolecular phase separation. It also summarizes advances in phase‑separation‑targeted therapeutic strategies and the design of functional biomaterials inspired by this process.

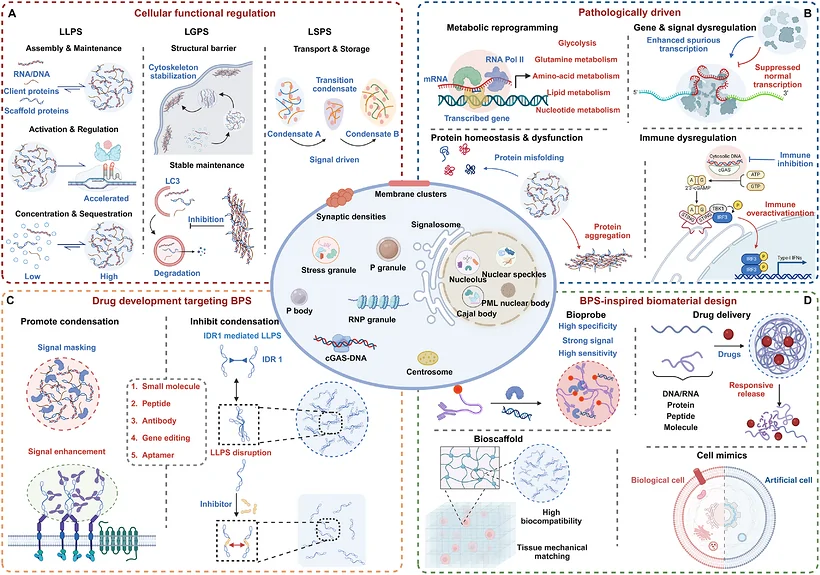

## 1 Introduction

Biomolecular phase separation (BPS) is defined as the spontaneous demixing behavior of biological molecules including proteins and nucleic acids [1, 2]. Such physical behaviors take place in intracellular environments or in vitro biological systems under specific conditions, generating independent phases with unique physicochemical properties that differ from uniform surrounding solutions [3, 4]. Early recognition of cellular membrane‐less compartments originated from the discovery of nucleoli in the 19th century, yet researchers at that time failed to identify the phase separation characteristics of these structures due to limited technical and methodological support [5]. In 2009, Hyman and Brangwynne pioneered the connection between phase separation theory and biological systems via their investigations of P granules in Caenorhabditis elegans [6, 7, 8]. Their landmark work laid vital conceptual and experimental foundations for subsequent studies on biological phase separation. Since then, renewed studies of membrane‐less organelles (MLOs), such as the nucleolus, ribosome‐related condensates, and stress granules, have further suggested that phase separation is a common intracellular principle involved in many essential biological processes [9, 10, 11]. In addition, the properties of BPS are not limited to the classical liquid state typically associated with liquid‐liquid phase separation (LLPS), but can also exhibit gel‐like features defined as liquid‐gel phase separation (LGPS) or solid‐like features defined as liquid‐solid phase separation (LSPS) [12, 13]. Dynamic transitions across the three phase separation modes precisely tune the aggregation state and spatial distribution of intracellular biomolecules [14, 15]. This regulatory process governs a spectrum of essential physiological activities, such as membrane‐less organelle assembly and function, signal transduction, biomolecular transport and storage, as well as cell cycle control [16, 17, 18]. Among these, LLPS is the most prevalent form, enhancing gene transcription efficiency by increasing the local concentration of transcription‐related molecules [19, 20, 21]. LGPS integrates dynamic flexibility with structural stability, assisting cells in coping with acute stress and functional transformation. LSPS primarily constructs stable rigid structures, such as intermediate filament networks, to reinforce cellular mechanical stability [22, 23, 24]. Collectively, these phase separation behaviors are essential for maintaining normal cellular physiological functions and intracellular homeostasis (Figure 1A).

**FIGURE 1:**
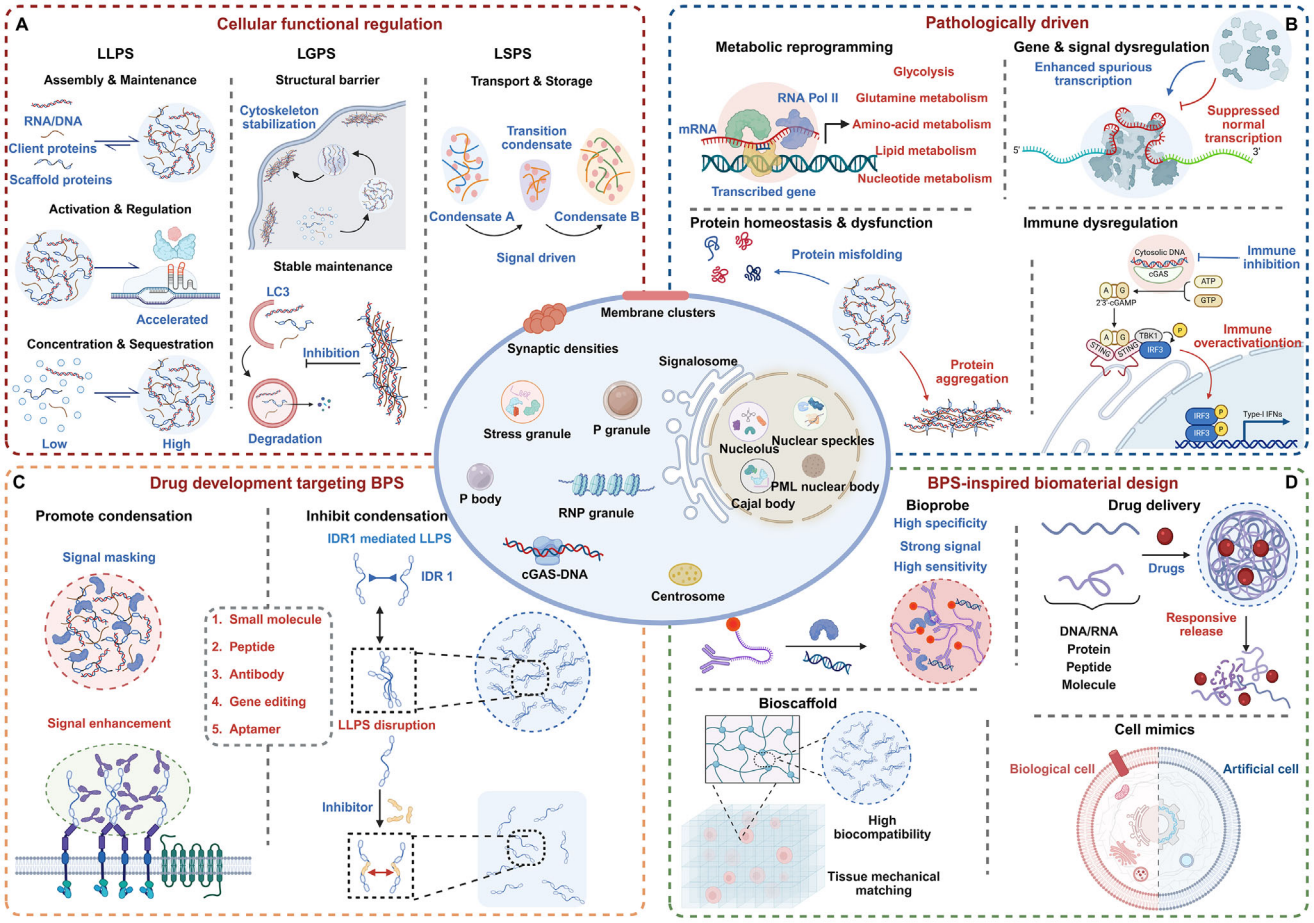
Schematic illustration of biomolecular phase separation in biomaterials design, highlighting its physiological roles, pathological dysregulation, and therapeutic opportunities. (A) Cellular functional regulation of BPS. (B) BPS induces disease pathogenesis. (C) Drug development targeting BPS. (D) BPS‐inspired biomaterial design.

Accumulating research findings verify that abnormal phase separation behaviors are closely correlated with the progression of multiple diseases (Figure 1B) [25, 26]. In neurodegenerative disorders, aberrant phase separation and aggregation of proteins represent key pathological hallmarks [27]. Mutated or misfolded proteins form insoluble aggregates in cells, and the accumulation of these aggregates leads to neuronal damage and death [28, 29, 30]. In cancer, phase separation is also involved in tumor initiation, progression, and metastasis. Proteins associated with oncogenes or tumor suppressor genes alter their normal functions and subcellular localization through aberrant phase separation, thereby disrupting cellular proliferation, differentiation, and apoptosis [31, 32]. Investigations into the mechanisms and functions of phase separation at the molecular level have revealed that BPS is gradually evolving from a biological concept into a manipulable therapeutic target [33, 34]. Traditional drug development primarily targets proteins with well‐defined three‐dimensional structures. However, many disease‐related proteins lack stable conformations, rendering them “undruggable”. Elucidating the mechanisms of BPS has made these previously untargetable targets feasible (Figure 1C) [35, 36, 37]. By regulating BPS processes, developing small molecules, nucleic acid aptamers, or peptides capable of modulating abnormal phase separation can intervene in disease‐related biological processes, offering novel strategies for drug design [38, 39].

Meanwhile, the characteristics exhibited by BPS‐including molecular self‐assembly, dynamic reversibility, and responsiveness to environmental stimuli offer rich design concepts for developing advanced intelligent biomaterials [40, 41]. From the materials science perspective, biomaterials engineered by mimicking BPS mechanisms are expected to exhibit superior performances and functionalities, such as high biocompatibility, precise targeting, intelligent responsiveness, and efficient loading/release capabilities (Figure 1D) [42]. These properties satisfy the stringent requirements for biomaterials in biomedical engineering, tissue engineering and drug delivery [43, 44]. In drug delivery, BPS‐induced nanomaterials can improve targeted delivery and allow controlled drug release [45, 46]. In tissue engineering, BPS‐inspired 3D scaffold materials can create a more suitable microenvironment for cell growth, proliferation, and differentiation, which in turn supports tissue repair and regeneration [47, 48, 49]. Interdisciplinary research on BPS spanning biology and materials science enables deeper insights into the nature of life phenomena.

Despite these significant advances, an integrated perspective that coherently bridges the spectrum of phase transitions, their pathological implications, and materials innovation remains conspicuously absent. Herein, our review will systematically examine the transitions and functional distinctions among these phase states, with particular emphasis on how their dynamic interconversion regulates both physiological and pathological processes. We will further clarify the direct correlations between the physicochemical rules of phase separation and the rational construction of high‐performance biomaterials. This work shifts research from simple phenomenological description to systematic exploration of the “property‐design‐function” paradigm grounded in fundamental physical and chemical principles. Based on the recent research progress, this review comprehensively summarizes biomaterials derived from biomolecular phase separation. It centers on therapeutic strategies for regulating abnormal phase separation via induction or suppression, alongside the development of innovative biomaterials such as cell‐mimetic systems, responsive drug vehicles, bioactive scaffolds and imaging probes. Finally, we will critically evaluate current technical challenges and outline viable translational pathways, offering a unified conceptual framework that bridges cell biology, pharmacology, and materials science.

## 2 Physiological Regulation of Biomolecular Phase Separation

BPS enables highly dynamic and tightly‐controlled molecular organization inside cells. This process starts mainly with LLPS, a reversible thermodynamic demixing process that produces biomolecular condensates bearing intrinsic liquid‐like properties. These initially liquid condensates may progress through a liquid‐gel‐solid continuum of material‐state transitions. Multivalent network crosslinking (gelation/percolation), ordered molecular stacking, and kinetic arrest drive these shifts and confer gel‐like or solid‐like characteristics to the assemblies [50, 51]. Critically, only LLPS represents a primary, thermodynamically driven de novo phase separation event. LGPS and LSPS do not constitute independent phase separation modes. Instead, they describe kinetically regulated material state maturation of condensates originally formed via LLPS. This conceptual distinction clarifies the boundary between thermodynamic phase separation itself and subsequent changes in condensate material properties including gelation, percolation, kinetic arrest, and liquid‐to‐solid fibrillization. Condensates across this state continuum assemble distinct functional compartments in cells that mediate key processes such as membraneless organelle formation, signal transduction, and metabolism, thereby maintaining intracellular homeostasis [52, 53]. A clearer understanding of the specific roles and regulatory mechanisms of different forms of phase separation in cells is therefore essential for elucidating fundamental biological processes. The compositional profiles and specialized biological functions of representative endogenous membraneless organelles are systematically sorted out in Table 1. As summarized within this table, all MLOs rely on intrinsically disordered proteins and functional RNA fragments as core scaffolding components to establish liquid‐like compartmentalized microenvironments, yet they differ sharply in accessory binding factors and targeted downstream biochemical reactions. For instance, nucleoli enriched with FBL, TERT and rRNA mainly undertake ribosome biogenesis, while stress granules assembled via G3BP and mRNA function as temporary repositories to sequester untranslated transcripts under oxidative stress. Such compositional differences directly determine their divergent physiological roles, which establishes a clear causal link between molecular building blocks and condensate bioactivity. It also provides a basis for further studies on the links between aberrant phase separation and disease, as well as for the development of biomaterials inspired by phase separation principles.

**TABLE 1:** Composition and functions of membraneless organelles [54, 55, 56, 57].

Condensate	Composition	Mechanism	Function
**Cajal body**	snRNA, snRNP proteins, RNA‐modifying enzymes, telomerase RNA	Hydrophobic interactions of Coilin protein LCDs and specific RNA‐protein binding drive phase separation	**1**. snRNA processing and snRNP assembly **2**. Telomerase RNA processing to maintain telomere length
**Cleavage body**	Nucleases, RNA polymerase II, pre‐mRNA, RNA‐binding proteins	Multivalent RNA‐protein interactions trigger phase separation	**1**. Participate in 3' end cleavage and polyadenylation of pre‐mRNA **2**. Regulate mRNA maturation
**DNA damage foci**	DNA damage repair proteins, histone variants, RNA (damage‐related lncRNA), RNF168 ubiquitin ligase	γH2AX‐mediated local enrichment of chromatin and multivalent interactions of DNA damage repair proteins drive phase separation	**1**. Protect damaged DNA from further damage **2**. Enrich DNA repair factors to improve repair efficiency **3**. Activate DNA damage stress pathways
**Gems**	snRNP proteins, telomerase protein, telomerase RNA, FBL	Self‐assembly of Coilin protein and RNA‐protein interaction of TERT‐TERC complex trigger phase separation	**1**. Participate in the maturation and storage of snRNP **2**. Assist in telomerase complex assembly and regulate telomere elongation **3**. Cooperate with Cajal bodies to maintain the homeostasis of nuclear RNA processing
**Heterochromatin**	Histones, heterochromatin proteins, DNA, SUV39H1 methyltransferase	Multivalent polymerization and hydrophobic interactions trigger phase separation	**1**. Mediate gene silencing **2**. Condense chromatin and maintain genomic structural stability **3**. Protect telomere regions and prevent chromosome end fusion
**Histone locus body**	Histone mRNA, RNA polymerase II, histone processing factors, transcription factors, lncRNA	Multivalent interactions of NPAT protein and RNA‐protein binding of histone mRNA drive phase separation	**1**. Regulate histone gene transcription **2**. Participate in histone mRNA processing **3**. Coordinate histone synthesis and cell cycle
**Nuclear speckle**	Spliceosome snRNP, splicing factors, pre‐mRNA, lncRNA	Charge interactions of RS domains of splicing factors and binding to pre‐mRNA splice sites trigger phase separation	**1**. Store and sort splicing factors **2**. Regulate alternative splicing of pre‐mRNA **3**. Participate in genome organization and link enhancers with promoter
**Nuclear stress body**	Heat shock factor 1, satIII RNA, RNA‐binding proteins, heat shock proteins	Stress induces HSF1 activation and binding to satIII RNA, triggering phase separation mediated by multivalent RNA‐protein interactions	**1**. Serve as a temporary storage pool for stress‐related RNA/proteins **2**. Protect nuclear proteins from stress damage **3**. Regulate the expression of heat shock genes and mediate cellular stress adaptation
**Nucleolus**	rDNA, rRNA, ribosomal proteins, nucleolar proteins, RNA polymerase I	Charge interactions between rRNA and ribosomal proteins, hydrophobic interactions of NPM1 LCDs form multiphase condensates	**1**. Ribosome biogenesis **2**. Perceive cellular stress **3**. Protein quality control
**p62 body**	p62/SQSTM1 protein, LC3, ubiquitinated proteins, Keap1, mTOR kinase	Oligomerization is mediated by the PB1 domain of p62 and binding of the UBA domain of ubiquitinated proteins drive phase separation	**1**. Mediate autophagic substrate recognition and degradation **2**. Participate in post‐translational regulation of proteins **3**. Respond to oxidative stress
**PML body**	PML protein, histone chaperone, SUMO‐modified proteins, p53, antiviral proteins	Multimerization mediated by the RING domain of PML and SUMO‐interacting motif interactions trigger phase separation	**1**. Mediate apoptotic signal transduction **2**. Antiviral defense **3**. Regulate transcription
**Transcriptional condensate**	Transcription factors, coactivators, RNA polymerase II, enhancer RNA, histone‐modifying enzymes	Hydrophobic interactions of LCDs in the activation domain of transcription factors and eRNA‐protein charge interactions trigger phase separation	**1**. Drive active transcription **2**. Coordinate the assembly of transcription factors, coactivators, and RNA polymerase II **3**. Regulate the expression of cell identity genes
**Balbiani body**	Mitochondria, endoplasmic reticulum fragments, RNA, RNA‐binding proteins, mitochondria‐related proteins	Multivalent interactions of RNA‐binding proteins and anchoring of mitochondrial membrane proteins drive phase separation	**1**. Store organelles and RNA in oocytes **2**. Protect and regulate germline‐related mRNA **3**. Maintain the meiotic arrest state of oocytes
**Nuclear pore complex**	Nucleoporins, RanGTPase, nuclear transport receptors, RNA	Hydrophobic interactions of FG‐repeat proteins and RanGTP‐dependent binding of nuclear transport receptors drive phase separation	**1**. Regulate nucleocytoplasmic transport **2**. Maintain the homeostasis of the nuclear environment **3**. Participate in transcriptional regulation
**Centrosome**	Centrosomal proteins, microtubule‐associated proteins, kinases, γ‐tubulin ring complex	Hydrophobic interactions of PCM1 protein LCDs trigger phase separation	**1**. MTOC regulate microtubule nucleation and stability **2**. Ensure equal chromosome segregation **3**. Maintain cell polarity, motility, and adhesion
**Paraspeckles**	Long non‐coding RNA, RNA‐binding proteins, pre‐mRNA, splicing factors	NEAT1 acts as a “scaffold” to bind PSPC1/NONO, triggering phase separation through multivalent RNA‐protein interactions	**1**. Regulate gene expression **2**. Store RNA‐binding proteins during stress to maintain cellular homeostasis **3. 3**. Participate in antiviral defense
**P‐body**	mRNA‐degrading enzymes, RNA‐binding proteins, untranslated mRNA, miRNA	Interactions between RNA‐binding protein LCDs and mRNA trigger phase separation	**1**. mRNA degradation to regulate mRNA half‐life **2**. Temporarily store mRNA with low translation efficiency **3**. miRNA‐mediated gene silencing
**P granule**	RNA, RNA‐binding proteins, histone chaperones, DEAD‐box helicases	RNA‐protein interactions and self‐assembly of PGL1/3 drive phase separation	**1**. RNA storage and sorting in germ cells of Caenorhabditis elegans **2**. Regulate the post‐transcriptional expression of germline‐related genes **3**. piRNA‐mediated transposon silencing to protect genomic stability
**Proteasome storage granule (PSG)**	26S proteasome, proteasome assembly factors, molecular chaperones, ubiquitinated proteins	Multivalent interactions of proteasome subunits and scaffolding effect of POMP drive phase separation	**1**. Store proteasomes under stress conditions to avoid degradation **2**. Release proteasomes after environmental recovery to quickly restore protein degradation function **3**. Participate in protein quality control and degrade misfolded proteins
**RNA transport granule**	mRNA, RNA‐binding proteins, microtubule motor proteins, ribosomal subunits	Specific binding of RNA‐binding proteins to mRNA triggers phase separation	**1**. Directionally transport specific mRNA **2**. Regulate the translation timing of mRNA **3**. Maintain cell polarity and functional specialization
**Stress granule (SG)**	Untranslated mRNA, RNA‐binding proteins, translation initiation factors, HSP70 family chaperones	Stress induces translation arrest, and LCDs of RNA‐binding proteins trigger phase separation through hydrophobic interactions and π‐π stacking	**1**. Protect mRNA from stress‐induced degradation **2**. Pause the translation of non‐essential genes to prioritize the expression of stress‐related genes **3**. May be involved in neurodegenerative diseases when abnormal
**U‐body**	U‐type snRNP, snRNP assembly factors, RNA‐binding proteins, P‐body associated proteins	Interactions between snRNP assembly factors and U‐type snRNA trigger phase separation	**1**. Participate in the assembly and storage of U‐type snRNP **2**. Assist in the transport of snRNP to Cajal bodies
**TIS granule**	Translation initiation factors, RNA‐binding proteins, ER‐associated mRNAs, ribosomal subunits	Multivalent interactions of translation initiation factors and the anchoring effect of ER membrane proteins drive phase separation	**1**. Regulates the translation of ER‐associated mRNAs **2**. Coordinates translation and protein translocation **3**. Maintains ER protein homeostasis and prevents mislocalization
**Signalling cluster**	Membrane receptors, signaling kinases, adapter proteins, phospholipids	Protein phosphorylation mediated by receptor activation triggers multivalent interactions and drives phase separation	**1**. Amplifies signal transduction **2**. Enhances signal specificity and reduces non‐specific activation **3**. Regulates immune responses, cell proliferation, and differentiation
**Synaptic density**	neurotransmitter receptors, scaffold proteins, signaling kinases, cell adhesion molecules	The PDZ domains of scaffolding proteins mediate multivalent interactions and trigger phase separation	**1**. Mediates synaptic signal transmission between neurons **2**. Regulates synaptic plasticity **3**. Maintains synaptic structural stability and participates in neural development and repair

LCD, Low‐complexity domain; snRNA, Small nuclear ribonucleic acid; snRNP, Small nuclear ribonucleoprotein; rRNA, Ribosomal ribonucleic acid; lncRNA, Long non‐coding ribonucleic acid; mRNA, Messenger ribonucleic acid; NPC, Nuclear pore complex; MTOC, Microtubule‐organizing center; PCM, Pericentriolar material; P‐body, Processing body; PSG, Proteasome storage granule; FG, Phenylalanine‐glycine; SUMO, Small ubiquitin‐like modifier; UBA, Ubiquitin‐associated domain; PB1, Phox and Bem1 domain; Keap1, Kelch‐like ECH‐associated protein 1; mTOR, Mechanistic target of rapamycin kinase; γH2AX, Phosphorylated histone H2AX; FBL, Fibrillarin; TERT, Telomerase reverse transcriptase; TERC, Telomerase RNA component; NPAT, Nuclear protein of the ataxia telangiectasia locus / Nuclear coactivator of histone transcription; PSPC1, Paraspeckle component 1; NONO, Non‐POU domain‐containing octamer‐binding protein; PGL, P granule protein; piRNA, PIWI‐interacting RNA; POMP, Proteasome maturation protein.

### 2.1 LLPS: Dynamic Compartmentalization in Cells

LLPS is one of the most common forms of BPS. It is characterized by the spontaneous demixing of biomolecules from a homogeneous solution under specific conditions through multivalent weak interactions, such as hydrophobic interactions, hydrogen bonding, and electrostatic interactions, leading to the formation of condensates with liquid‐like properties [58]. At the molecular level, this process follows the canonical sticker‐spacer framework [59]. Folded binding domains or short linear motifs act as adhesive “stickers” to mediate specific intermolecular contacts, while IDRs serve as flexible “spacers” that preserve solubility and dynamic molecular exchange. The valency and affinity of stickers, together with charge patterning, aromatic residue distribution and hydrophobic content of IDR sequences, collectively define key phase parameters including saturation concentration, condensate viscosity and internal molecular turnover rate. These multivalent interaction systems can be quantitatively described by extended Flory‐Huggins polymer thermodynamics, which characterizes the free‐energy balance that drives demixing from bulk solution [60]. Computational and theoretical approaches have further deepened mechanistic understanding of BPS. Coarse‐grained molecular dynamics simulations recapitulate mesoscale phase separation dynamics and quantify how interaction valency shapes phase boundaries [61]. All‐atom simulations resolve residue‐level interaction networks within IDRs, clarifying how pathogenic mutations alter phase behavior at the molecular scale. AI‐powered sequence‐based prediction models have also emerged as high‐throughput tools, enabling identification of putative phase‐separating proteins and rational design of molecules with tailored phase transition profiles. These condensates can respond to the intracellular environment changes through behaviors such as fusion and fission [62, 63]. Meanwhile, they can significantly increase the concentration of functional molecules in local regions, providing a unique microenvironment for efficient biochemical reactions and signal transduction within cells [64]. In cellular activities, LLPS ensures the precise execution of cellular physiological functions mainly through three major pathways: participating in the formation of MLOs, regulating the efficiency of signal transduction, and enabling the concentration and sequestration of substances [65, 66].

#### 2.1.1 Assembly and Homeostasis of Membraneless Organelles

In the formation and functional maintenance of MLOs, the nucleolus and stress granules (SGs) serve as representative examples regulated by LLPS [67, 68]. The nucleolus achieves LLPS via multivalent interactions between intrinsically disordered regions of resident proteins and RNAs [69, 70]. This process concentrates key factors for rRNA synthesis and ribosomal subunit assembly, supporting ribosome biogenesis and dynamically adjusting ribosome production under stress to accommodate environmental shifts [71, 72]. SGs, on the other hand, assemble rapidly via LLPS under heat shock, viral infection, or oxidative stress, through the co‐condensation of untranslated mRNAs with RNA‐binding proteins [73, 74, 75]. They sequester mRNAs to prevent degradation while activating cytoprotective responses via signaling pathway interactions, and reversibly disassemble upon stress relief to release mRNAs for normal translation [76, 77]. These dynamic regulatory processes highlight the central role of LLPS in cellular stress adaptation and functional homeostasis maintenance.

#### 2.1.2 Activation and Regulation of Cellular Signal Transduction

LLPS plays dual roles as a “signal amplifier” and a “regulatory switch” in cellular signal transduction. By concentrating signaling molecules in specific regions, LLPS enables the rapid transmission and precise regulation of signals [78]. As illustrated in Table 1, multiple signaling‐related membraneless compartments such as P‐bodies dynamically assemble to coordinate post‐transcriptional signal transduction. Taking the cellular adaptive response to heat shock as an example, phosphorylated heat shock transcription factor 1 (HSF1) undergoes LLPS via its intrinsically disordered regions, forming functional condensates at target gene loci (Figure 2A) [79]. These condensates efficiently recruit the transcriptional machinery to activate heat shock protein expression, thereby facilitating protein folding and maintaining proteostasis. By shortening molecular diffusion distances and enhancing interaction probabilities, this process enables cells to complete a stress response within minutes, markedly improving signal transduction efficiency. In addition, LLPS is also involved in the regulation of processes such as cell cycle progression and growth factor signaling [78, 80, 81]. Kinases including cyclin‐dependent kinase 1 (CDK1) phosphorylate intrinsically disordered repetitive sequences of Ki‐67 during mitosis. This modification elevates the protein's LLPS tendency and redistributes Ki‐67 from the nucleolus to mitotic chromosome peripheries to assemble the perichromosomal layer (Figure 2B) [82]. This structure prevents aberrant chromosome condensation and guarantees the normal progression of mitosis. During Synthesis phase (S phase), LLPS allows Ki‐67 to build a protective microenvironment around DNA replication forks and preserve genome stability. In the epidermal growth factor (EGF) signaling cascade, activated receptors recruit adaptors including growth factor receptor‐bound protein 2 (Grb2) and son of sevenless (SOS); multivalent interactions among these proteins drive the assembly of membrane‐tethered signaling condensates [83, 84]. These assemblies restrict molecular diffusion and augment local signal strength, enabling precise regulation of cell proliferation and differentiation. Notably, membrane‐tethered signaling condensates follow a distinct two‐dimensional phase separation regime, unlike three‐dimensional cytoplasmic LLPS. The planar membrane interface restricts molecular diffusion to two dimensions, lowering the critical concentration for phase separation and allowing rapid assembly of signaling complexes at nanomolar physiological concentrations [85, 86]. Membrane phospholipids such as phosphatidylinositol 4,5‐bisphosphate (PIP_2_) act as multivalent anionic scaffolds, recruiting positively charged signaling proteins via electrostatic interactions and synergistically promoting membrane‐bound phase separation [87]. A prototypical example appears in T cell receptor (TCR) signaling. Upon antigen stimulation, the adaptor protein LAT assembles into liquid‐like signaling clusters on the inner plasma membrane through multivalent interactions with downstream adaptors and kinases [88]. These membrane condensates amplify proximal signaling while excluding negative regulatory phosphatases, securing the specificity and sensitivity of immune activation.

**FIGURE 2:**
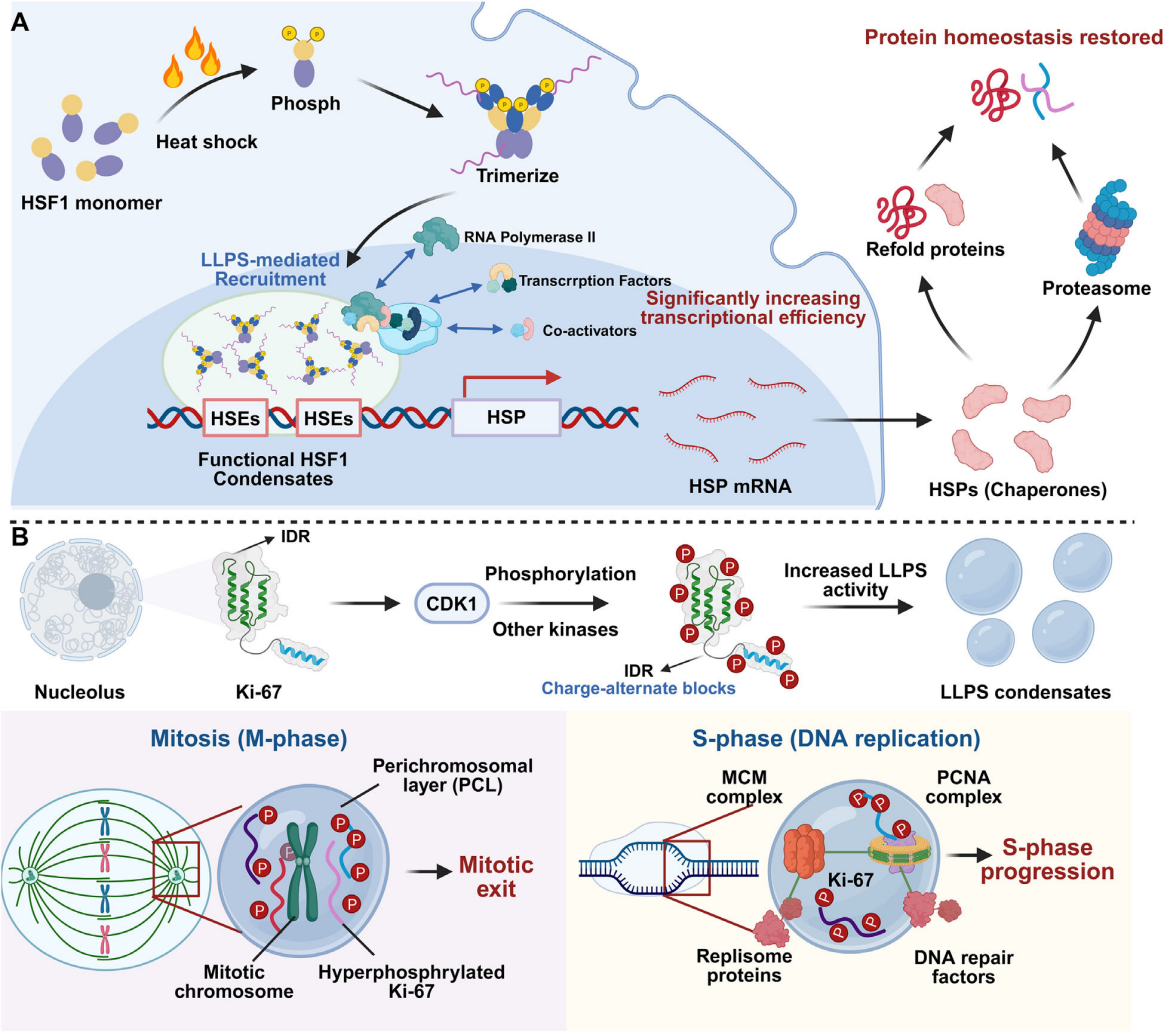
LLPS‐induced cellular physiological processes regulation. (A) LLPS drives HSF1 condensates formation to enhance heat shock proteins transcription under heat shock. (B) Ki‐67 phosphorylation alters its charge distribution and LLPS propensity to control its localization and function across S phase and mitosis.

Beyond intracellular membrane‐proximal condensation, phase separation also occurs at the extracellular cell surface to orchestrate growth factor signaling and cell‐matrix communication. Basic fibroblast growth factor (bFGF), a key regulator of cell proliferation and tissue repair, undergoes multivalent electrostatic interactions with heparan sulfate proteoglycans on the plasma membrane to form liquid‐like condensates [89]. These cell‐surface condensates concentrate bFGF ligands and promote co‐clustering of fibroblast growth factor receptors, thereby strengthening downstream Ras‐ERK and PI3K‐Akt signal transduction. Similar surface phase separation mechanisms have also been identified in immune cell adhesion and postsynaptic transmission: integrin‐based focal adhesions and synaptic density assemblies both rely on transmembrane and extracellular phase separation to mechanically couple extracellular cues with intracellular cytoskeletal and signaling dynamics [90, 91]. Collectively, these findings expand the physiological scope of BPS from intracellular compartments to cell‐surface microdomains, highlighting its universal role in spatial signal organization across cellular boundaries.

#### 2.1.3 Concentration and Sequestration of Biochemical Reaction Substrates

There are two mechanisms by which LLPS regulates intracellular biochemical reactions. First, it concentrates key enzymes and substrates to boost reaction kinetics. Second, it sequesters reactive components to block unwanted nonspecific interactions and sustain the ordered arrangement of intracellular metabolic networks [92, 93, 94]. The process of DNA replication is a representative example of such regulatory effects. Replication‐associated proteins (such as DNA polymerases, helicases and primases) engage in multivalent binding with fork‐associated DNA and various cofactors at active replication forks [95, 96]. These intermolecular interactions trigger LLPS at replication sites to assemble concentrated replisome condensates, which elevate local replication factor concentration roughly 10‐ to 100‐fold to strengthen protein‐protein cooperativity and accelerate DNA synthesis [97, 98]. In vitro research further verifies that modulation ionic strength can trigger LLPS in replication‐associated proteins. Compared with homogeneous reaction systems, such phase‐separated systems improve DNA replication efficiency around 3‐ to 5‐fold [99].

The activation of inflammasomes represents a critical host response to pathogen invasion in innate immunity. Precise tight control is required to limit excessive inflammation and collateral damage to host cells [100, 101]. As a key regulator of cell death and inflammatory signaling, receptor‐interacting serine/threonine‐protein kinase 1 (RIPK1) triggers excessive inflammatory responses upon aberrantly activation. Inflammasome‐associated proteins (such as PARP5A and RNF146) can form dynamic liquid‐like condensates through LLPS [102, 103]. These condensates selectively recruit and modify activated RIPK1 to sequester this RIPK1 and downstream signaling effectors into defined subcellular regions. This sequestration limits aberrant crosstalk with other signaling pathways and prevents nonspecific inflammatory activation [74]. Accordingly, pathogen stimulation triggers rapid yet spatially constrained inflammatory signaling transduction to enable precise immune responses [104].

### 2.2 LGPS: Structural Support and Intracellular Trafficking

LGPS describes the intermediate material state formed when liquid condensates (generated via initial LLPS) undergo liquid‐to‐gel transition through progressive multivalent crosslinking and network percolation. This transition is typically driven by enhanced intermolecular interactions, post‐translational modifications, or shifts in environmental conditions, which transform dynamically fluid droplets into viscoelastic gel‐like assemblies [105]. Unlike the thermodynamic equilibrium of LLPS, gelation is often a kinetically controlled process that builds a three‐dimensional crosslinked network within condensates. This gel phase possesses higher mechanical strength and structural stability alongside diminished internal molecular mobility relative to liquid LLPS droplets. It still retains moderate molecular permeability and partial reversibility under physiological conditions. In cellular systems, LGPS mainly participates in cellular structure assembly and substance transport and storage regulation, providing physical support to cells and sustains metabolic homeostasis [106, 107]. By balancing the diffusion tendency of liquids and the excessive rigidity of solids, LGPS endows cells with adaptive capacity to environmental changes while preserving cellular morphology and function [108].

#### 2.2.1 Construction of Cytoskeleton and Mechanical Homeostasis

LGPS participates in the assembly and functional maintenance of actin networks and microtubule systems, balancing the structural stability and dynamic plasticity of the cytoskeleton [109, 110]. Taking actin network assembly as an example, actin monomers are initially enriched into high‐concentration condensates via LLPS, which accelerates nucleation [111, 112]. Subsequently, under the regulation of actin‐binding proteins (such as actin‐binding LIM protein 1 (abLIM1)), the monomers gradually polymerize into filaments, which are further crosslinked into dense three‐dimensional gel networks that constitute the cortical cytoskeleton [112, 113]. For instance, the actin gel layer beneath the apical membrane of epithelial cells resists mechanical stress and maintains cell morphology [114]. This gel structure remains dynamically responsive to external mechanical stimuli and sustains mechanical homeostasis through reversible phase transitions. Under tensile stress, conformational changes in crosslinking proteins modulate their binding affinity, allowing reversible deformation of the gel to buffer mechanical stress [115]. Upon stress removal, the gel rapidly recovers its original structure, thereby preventing cytoskeletal damage [116]. This deformation‐recovery mechanism enables cells to adapt to physiological mechanical fluctuations, highlighting the central role of liquid‐gel phase separation in cellular mechanical adaptation.

#### 2.2.2 Regulation of Intracellular Substance Transport and Storage

LGPS refers to biomolecular condensates formed via liquid‐gel transition. This state controls cargo aggregation and release to coordinate the spatiotemporal organization of intracellular substance metabolism. SGs serve as a classical model for studying phase separation [75]. Under stresses like oxidative injury, viral infection and heat shock, RNA‐binding proteins G3BP1 and Caprin‐1 trigger condensate assembly via multivalent weak interactions of their intrinsically disordered regions [117, 118]. These liquid condensates accumulate untranslated mRNAs and translation initiation factors to facilitate stress granule biogenesis. Upon recruitment into SGs, WD repeat domain 45 (WDR45) interacts with Caprin‐1 to restrain the liquid‐to‐rigid transition of SGs cores, thereby maintaining mRNA storage and homeostasis (Figure 3).

**FIGURE 3:**
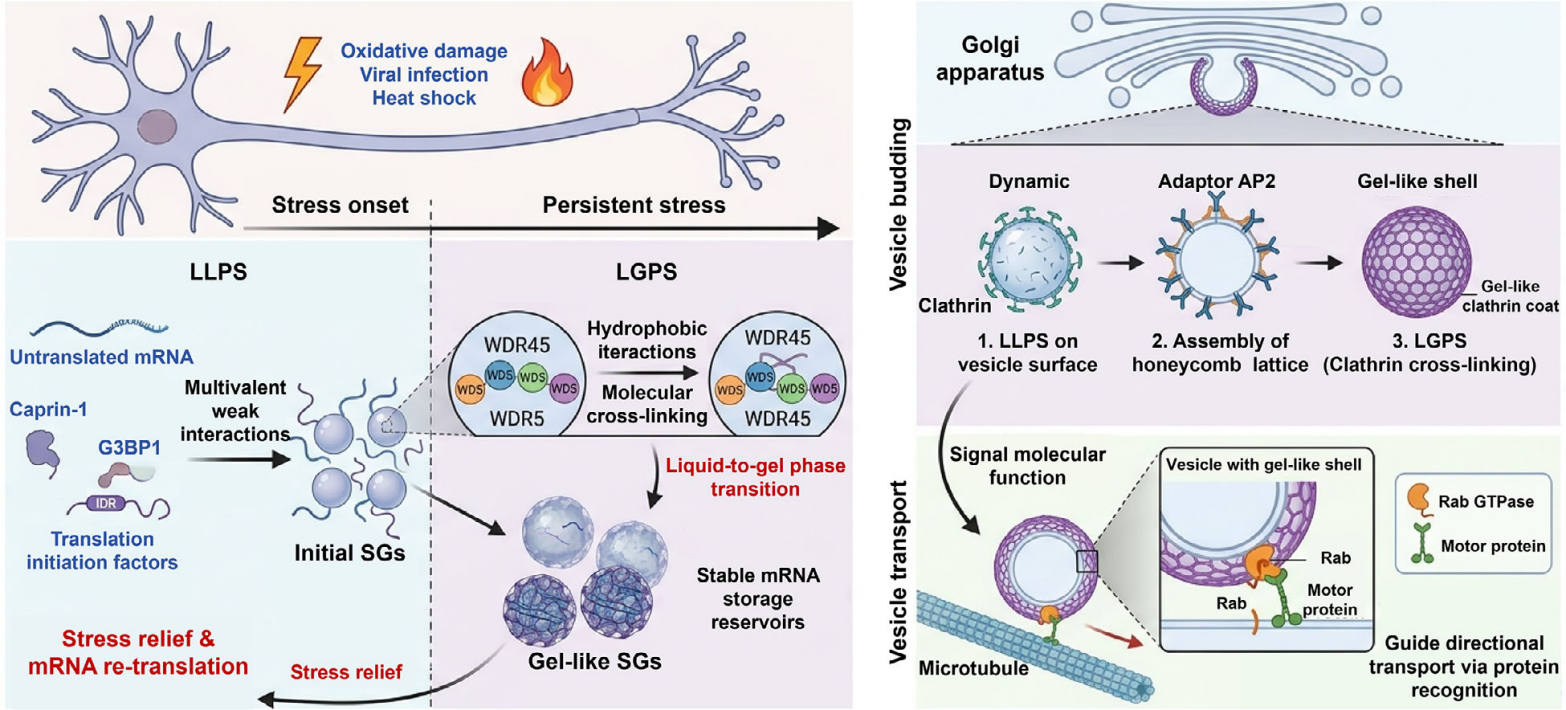
LGPS mediates the assembly of cytoskeletal networks to maintain cellular mechanical stability, and regulates the formation, material storage and directional transport function of stress granules and intracellular vesicles.

In intracellular transport, LGPS‐related assemblies ensure transport fidelity by modulating the stability and mechanical properties of transport carriers. Early endocytic proteins such as Eps15 and Fcho2 undergo LLPS‐like assembly on membrane surfaces, recruiting clathrin and adaptor proteins to endocytic sites [119]. Adaptor protein complex 2 (AP2), upon binding to membrane phosphatidylinositol 4,5‐bisphosphate and cargo molecules, undergoes conformational rearrangement that promotes clathrin polymerization into a honeycomb‐like lattice [120]. This lattice cooperates with membrane lipids to establish a stable protein‐lipid gel‐like interface, enhancing vesicle mechanical stability and reducing the risk of rupture during transport. Additionally, Ras‐related in brain guanosine triphosphatases (Rab GTPases) anchored on vesicle surfaces act as molecular switches, mediating specific recognition between vesicles and motor proteins to direct their targeted transport [121, 122]. Phase separation also underpins endosomal cargo sorting and membrane remodeling. The endosomal sorting complex required for transport (ESCRT) undergoes liquid‐liquid phase separation on endosomal membranes, driven by multivalent interactions between its subunits and ubiquitinated cargo proteins [123]. These membrane‐associated condensates concentrate ubiquitylated membrane proteins and drive intraluminal vesicle budding within multivesicular bodies. This mechanism enables precise sorting of cargoes, either for lysosomal degradation or recycling back to the plasma membrane. Unlike rigid membrane coat structures, phase‐separated ESCRT assemblies operate dynamically, matching the heterogeneous and rapidly changing nature of endosomal cargo processing.

### 2.3 LSPS: Stabilization of Cellular Physiological Structures

LSPS describes the terminal material state of condensate maturation, in which liquid or gel condensates undergo liquid‐to‐solid transition via kinetic arrest, ordered molecular stacking, or amyloid fibrillization. This process is typically driven by persistent stress, pathogenic mutations, or prolonged molecular aggregation. It transforms dynamic condensates into stable solid‑like assemblies that feature extremely low molecular mobility and limited internal molecular exchange [72, 124]. Unlike the reversible thermodynamic LLPS process, solidification is often irreversible under physiological conditions and represents a kinetically trapped end state of condensate maturation. In physiological contexts, controlled LSPS constructs stable structural assemblies such as the epidermal stratum corneum. In pathological settings, aberrant LSPS drives the formation of toxic insoluble protein aggregates. During epidermal differentiation, the formation of the stratum corneum relies on the ordered LSPS‐mediated assembly process [125]. In differentiating keratinocytes, profilaggrin/filaggrin‐associated factors initially assemble into keratohyalin granules via LLPS. Subsequently, the assembly and maturation of keratin‐associated components within these condensates are driven by the differentiation cues, accompanied by the liquid‐to‐solid phase transition [126, 127]. This ordered phase transition guarantees the structural stability of the stratum corneum while endowing robust skin barrier function. It enables the epidermis to resist external environmental insults and sustain intracellular homeostasis. Furthermore, LSPS‐mediated solid‐like structures enable efficient sequestration and controlled release of biomolecules, supporting the long‐term intracellular storage [128]. For example, glycogen can be stored in dense granule‐like structures when single‐celled organisms like yeast enter a dormant state. These granules shield glycogen from unintended degradation throughout cellular dormancy [129]. Following reactivation, specific enzymes gradually mobilize these reserves to rapidly replenish energy and guarantee seamless progression from dormancy to proliferative growth.

This chapter establishes a unified continuum framework spanning LLPS, LGPS and LSPS, and delineates their respective roles in core cellular processes. Yet most mechanistic insights stem from simplified in vitro reconstitution, which cannot replicate the crowded, chemically dynamic native intracellular environment. The causal hierarchy of multivalent interactions that govern phase state switching in living cells remains poorly defined, and few studies have quantified how mechanical forces or metabolic fluctuations dynamically remodel condensate material properties. Future work must integrate in situ biophysical characterization with functional genetic manipulation to move beyond phenomenological description and clarify the true physiological relevance of phase separation.

## 3 Pathological Dysregulation of Aberrant BPS

The dynamic continuum of LLPS, LGPS and LSPS operates under tight spatiotemporal control to sustain cellular homeostasis, with phase states precisely tuned to match functional demands across physiological contexts [130, 131]. This delicate equilibrium is vulnerable to disruption by genetic mutations, sustained environmental stressors such as oxidative stress and abnormal metabolite accumulation, and aberrant post‐translational modifications of core scaffolding proteins. Under such perturbations, physiological liquid condensates may undergo aberrant hardening into irreversible solid aggregates; gel‐like assemblies can lose adaptive plasticity and become rigid; and normally transient condensates may persist abnormally or mislocalize to ectopic subcellular compartments [132] (Table 2). These derailed phase transitions in turn drive four interconnected pathological cascades: protein homeostasis failure and toxic aggregation, dysregulated gene expression and signal transduction, organelle dysfunction and metabolic reprogramming, and impaired cellular stress adaptation and immune homeostasis [50]. This chapter systematically dissects how aberrant BPS drives the initiation and progression of diverse diseases by modulating these key biological processes.

**TABLE 2:** Corresponding Transition from Physiological BPS to Pathological Aberrant Phase Transitions.

Type	Physiological Homeostatic Functions	Perturbation Triggers	Pathological Phase Transition Process	Downstream Disease Manifestations
LLPS	1. Dynamic assembly of membraneless organelles (nucleolus, stress granules) 2. Local enrichment of signaling molecules for pathway amplification 3. Condensation of replication/transcription complexes to enhance efficiency	Pathogenic mutations, persistent oxidative stress, aberrant phosphorylation, RNA imbalance	1. Over‐stabilization of liquid droplets with impaired disassembly 2. Premature and irreversible liquid‐to‐solid transition 3. Ectopic formation of transcriptional condensates	1. Neurodegenerative diseases (solidification of TDP‐43/FUS stress granules) 2. Cancer (sustained oncogene activation by aberrant β‐catenin/YAP transcriptional condensates) 3. Autoimmune diseases (persistent inflammatory activation by cGAS‐DNA droplets)
LGPS	1. Cytoskeletal crosslinking to maintain mechanical homeostasis 2. Reversible mRNA storage in stress granules 3. Vesicle gel coats ensuring intracellular trafficking	Persistent stress, aberrant protein crosslinking, WDR45 loss‐of‐function	1. Irreversible hardening of the gel state 2. Disruption of vesicle gel coat structure and trafficking dysfunction 3. Impaired degradation of gelated stress granules	1. Neuronal axonal transport defects 2. Tumor chemoresistance (persistent sequestration of tumor‐suppressive mRNAs in stress granules)
LSPS	1. Epidermal stratum corneum barrier formation 2. Stable storage of metabolites in dormant cells	Protein misfolding, persistent metabolic stress	Transition from physiologically controlled solid structures into toxic insoluble protein fibrils	Alzheimer's disease (Tau/Aβ fibril deposition), skin keratinization disorders

### 3.1 Protein Homeostasis and Functional Dysregulation

The proper folding, assembly, transport and degradation of proteins collectively maintain intracellular protein homeostasis, which is fundamental to the execution of normal cellular physiological functions. BPS exerts vital regulatory effects on protein folding and degradation. Physiologically, biomolecules primarily undergo LLPS to generate dynamic, reversible condensates that locally concentrate proteins and support their correct folding and turnover. Condensates arising from LLPS can undergo subsequent shifts in material properties under pathological conditions. Such material remodeling does not inevitably yield insoluble protein aggregates. Depending on the protein species, post‑translational modifications and cellular microenvironment, dysregulated condensates may simply exhibit altered dynamics or mislocalize to ectopic subcellular compartments. They may also disturb cellular function through aberrant sequestration of partner molecules. In other circumstances, condensates may gradually develop gel‑like or solid‑like character. The transition into irreversible, cytotoxic insoluble aggregates or amyloid fibrils represents only one possible pathological outcome. Consequently, disturbed BPS may drive protein homeostasis imbalance via diverse routes, and aggregate formation is not an obligate consequence. Table 3 systematically catalogs a wide range of disease‐associated proteins and signaling factors linked to aberrant BPS. From the molecular information compiled in this table, neurodegenerative diseases mostly arise from the irreversible liquid‐to‐solid maturation of RNA‐binding protein condensates. Tumor development, by contrast, is primarily propelled by over‐stabilized transcriptional assemblies with modified phase transition kinetics. This table records two major divergent pathological patterns of biomolecular condensation across various disease types.

**TABLE 3:** Disease‐associated proteins and signaling factors regulated by dysregulated BPS [30, 38, 133].

	Proteins/Signaling factors	Disease type	Mechanism
Neurodegenerative Diseases Neurodegenerative Diseases	β‐amyloid	Alzheimer's disease (AD)	β‐amyloid oligomers accelerate the conversion of droplets into irreversible amyloid fibrils through the phase separation process
	Tau	Alzheimer's disease (AD)	Tau protein oligomers drive amyloid fibril formation through phase separation, lose normal function of stabilizing microtubules
	U1‐70K	Alzheimer's disease (AD)	U1‐70K undergoes nucleotide‐independent phase separation to transform into insoluble aggregates, and colocalizes with Tau neurofibrillary tangles
	APP_V225A_	AD	APP_V225A_ enhances binding affinity to Tau protein, exacerbating Tau phosphorylation and phase separation
	α‐synuclein	PD	α‐Synuclein undergoes phase separation mediated by its N‐terminal lipid‐binding domain and IDR, and the formed amyloid fibrils deposit as Lewy bodies
	TDP‐43	ALS, FTD	The RNA‐binding domains and low‐complexity domain of TDP‐43 mediate phase separation to form physiological stress granules, which subsequently undergo hardening into irreversible pathological aggregates
	FUS	ALS	FUS mediates phase separation through its N‐terminal low‐complexity domain, while ALS‐associated mutations accelerate the liquid‐to‐solid phase transition of condensates into fibrillar aggregates
Cancer Cancer	p53/53BP1	Pan‐cancer	Cancer‐associated p53 mutations disrupt the folding of its core domain, accelerate the aggregation of condensates formed by p53 and 53BP1, impede DNA repair, and promote the malignant progression of cancer cells
	Wnt	Pan‐cancer	Wnt signaling activation induces β‐catenin to form transcriptionally active phase‐separated condensates in the nucleus, which sustainably activates the expression of downstream pro‐oncogenes
	SHP2	Pan‐cancer	SHP2 modulates the MAPK signaling cascade via biomolecular phase separation and facilitates the activation of downstream ERK1/2 This molecular event strengthens proliferative and anti‐apoptotic signaling, thereby sustaining the malignant proliferation and survival of tumor cells
	YAP	Pan‐cancer	YAP fusion proteins undergo phase separation to enrich transcriptional co‐activators such as BRD4 and TEAD, while excluding transcriptional repressor complexes like PRC2, thereby sustainably activating pro‐oncogenic transcriptional programs
	FOXP1	Pan‐cancer	FOXP1 induces phase separation to form condensates at super‐enhancers. Through driving the transcription of downstream target genes such as SESN3 and regulating SP8 expression, it enhances the chemoresistance of cancer cells
	SRSF	Pan‐cancer	SRSF proteins undergo phase separation to regulate the aberrant alternative splicing of genes such as SFTA2, NUMB, and SLC37A4, generating pro‐oncogenic isoforms and activating related oncogenic signaling pathways including NOTCH
	STAT3	Pan‐cancer	IL‐6 triggers the tyrosine phosphorylation of STAT3 and further facilitates the formation of STAT3 biomolecular condensates via phase separation. This process enables STAT3 to efficiently modulate the transcription of target genes responsible for cell proliferation, anti‐apoptosis and tumor angiogenesis
	WDR3	Osteosarcoma	In osteosarcoma cells, WDR3 mediates phase separation via IDRs, forming condensates. Aberrations in its phase separation drive the malignant progression of osteosarcoma
	ILF3	Esophageal Cancer, Non‐Small Cell Lung Cancer	LncRNA MALR binds to the dsRBD1 domain of ILF3 and promotes phase separation. This process can prevent the degradation of HIF1α mRNA, and simultaneously participate in activating c‐Myc, thereby activating relevant oncogenic signaling pathways
	EGFR	Lung Cancer	EGFR mediate the phase separation process via NOVA protein, recruit DNA‐dependent protein kinase to repair DNA damage, and regulate the tumor‐promoting activation of the Ras signaling pathway. Meanwhile, its mutants can drive the activation of mTORC1 through phase separation‐related abnormal activation, thereby continuously activating downstream oncogenic signaling pathways such as MAPK and PI3K/Akt
	TOP2A	Liver Cancer	Aberrant activation of PKR kinase leads to its localization in the intrinsically disordered region of TOP2A. This process promotes the phase separation of TOP2A, facilitates DNA damage repair, and concurrently inhibits cGAS‐STING immune signaling pathway activation
	RIOK1	Liver Cancer	RIOK1 is transcriptionally upregulated by stress‐activated NRF2. It recruits IGF2BP1 and G3BP1 via phase separation to form stress granules, which sequester PTEN mRNA and inhibits its translation. Consequently, this process activates the pentose phosphate pathway and suppresses oxidative damage
	AR	Castration‐Resistant Prostate Cancer	Upon androgen stimulation, AR takes the NTD as the main driving force, cooperating with the DBD and LBD to undergo phase separation. These condensates enrich transcription‐related components and further activate the transcription of oncogenes
	cGAS	Systemic Lupus Erythematosus, Aicardi‐Goutières Syndrome	Upon binding to dsDNA, cGAS undergoes phase separation, which inhibits the degradation of immunostimulatory DNA, sustains the activation of the downstream STING pathway, and triggers excessive inflammatory responses
Autoimmune diseases	LAT	Combined Immunodeficiency	The abnormal phase separation of LAT leads to the excessive activation or dysregulation of its downstream signaling pathways, thereby triggering the hyperactivation or dysfunction of immune cells
	SLP65	B‐lymphoblastic leukemia	Upon activation of the B cell receptor by antigens, SLP65 undergoes three‐phase partitioning with CIN85 and lipid vesicles. The abnormality of this phase separation process impairs the immune function of B cells
	NLRP3	Chronic prostatitis, Familial cold autoinflammatory syndrome	NLRP3 undergoes phase separation with the IDR of FISNA. It recruits ASC to assemble the inflammasome and activate Caspase‐1, which leads to the continuous massive release of pro‐inflammatory cytokines such as IL‐1β, thereby triggering excessive inflammation and pyroptosis

AD, Alzheimer's disease; PD, Parkinson's disease; ALS, Amyotrophic Lateral Sclerosis; FTD, Frontotemporal Dementia; p53, Tumor protein p53; 53BP1, p53‐binding protein 1; ERK1/2, Extracellular signal‐regulated kinase 1/2; MAPK, Mitogen‐activated protein kinase; YAP, Yes‐associated protein; BRD4, Bromodomain‐containing protein 4; TEAD, TEA domain transcription factor; PRC2, Polycomb repressive complex 2; SRSF, Serine/arginine‐rich splicing factor; NOTCH, Neurogenic locus notch homolog protein; STAT3, Signal transducer and activator of transcription 3; IL‐6, Interleukin‐6; HIF1α, Hypoxia‐inducible factor 1 alpha; c‐Myc, cellular‐myelocytomatosis oncogene; EGFR, Epidermal growth factor receptor; mTORC1, Mechanistic target of rapamycin complex 1; PI3K/Akt, Phosphatidylinositol 3‐kinase / Protein kinase B; TOP2A, Topoisomerase DNA II alpha; PKR, Protein kinase R; NRF2, Nuclear factor erythroid 2‐related factor 2; IGF2BP1, Insulin‐like growth factor 2 mRNA‐binding protein 1; PTEN; Phosphatase and tensin homolog; AR; Androgen receptor; dsDNA, Double‐stranded deoxyribonucleic acid; SLE, Systemic Lupus Erythematosus; LAT; Linker for activation of T cells; NLRP3; NOD‐like receptor protein 3; ASC, Apoptosis‐associated speck‐like protein containing a CARD; IL‐1β, Interleukin‐1 beta.

#### 3.1.1 Pathological Protein Aggregation and Toxicity

Under physiological conditions, proteins can assemble into dynamic functional condensates via LLPS to participate in signal transduction and metabolism, and disassemble promptly after functional execution [134]. However, pathological triggers such as pathogenic gene mutations, persistent oxidative stress and aberrant post‐translational modifications can alter condensate material properties and slow or block disassembly. In specific protein systems (notably RNA‐binding proteins linked to neurodegeneration), these perturbations promote progressive liquid‐to‐gel or liquid‐to‐solid condensate maturation. In some cases, this maturation process culminates in the formation of insoluble, amyloid‐like aggregates [135]. Such aggregates lose physiological function, accumulate intracellularly or extracellularly, induce cytotoxicity and disrupt cellular homeostasis. As noted above, this transition to irreversible aggregation is not a universal outcome of dysregulated BPS. Many pathological condensate defects involve altered dynamics, mislocalization or aberrant sequestration of binding partners rather than insoluble aggregation.

In Alzheimer's disease (AD), phase separation of Aβ and Tau has been proposed to promote the formation of insoluble pathological deposits. While, the causal relationship between LLPS and in vivo amyloid formation remains incompletely resolved [136]. For Aβ, LLPS has been documented primarily for specific oligomeric species under defined in vitro conditions. Phase‐separated droplets can locally enrich Aβ molecules and accelerate nucleation of amyloid fibrils, yet LLPS is not established as a universal prerequisite for extracellular senile plaque formation in vivo. Aβ is generated from proteolytic processing of amyloid precursor protein (APP), and pathogenic mutations or dysregulated protease activity promote its extracellular deposition into senile plaques [137]. For Tau protein, aberrant post‐translational modifications promote its detachment from microtubules and enhance its phase separation propensity; liquid‐to‐solid maturation of Tau condensates is thought to contribute to the formation of intracellular neurofibrillary tangles [138, 139]. Together, these Aβ and Tau lesions damage neurons and drive AD progression.

#### 3.1.2 Protein Homeostasis Imbalance

Dysregulated BPS disrupts physiological protein folding and clearance pathways to trigger proteostasis defects and ectopic accumulation of insoluble protein aggregates. The protein homeostasis disruption is closely related to tumor development [140]. For instance, p53 as a central tumor suppressor is tightly regulated under normal conditions, controlling cell cycle progression to initiate apoptosis and coordinate cellular stress [141]. Cumulative evidence demonstrates that p53 engages in phase separation‐mediated assemblies to facilitate DNA damage repair and cellular stress adaptation (Figure 4) [142, 143]. Upon functional termination, p53 is physiologically degraded via the ubiquitin‐proteasome system (UPS) to sustain control over its intracellular abundance. However, this homeostatic balance is disrupted in hepatocytes acquiring cancer‐related TP53 mutations (e.g., R175H and G245S) or exposed to persistent inflammatory and oxidative stress [144, 145]. Under these conditions, the assembly behavior of p53 may shift from dynamic functional organization toward aberrant condensation with altered material properties and kinetics. Cancer‑associated p53 mutations modify its phase‑separation propensity in a mutation‑specific manner. Some mutant forms retain soluble functional assemblies with impaired activity, while others promote the formation of more stable or insoluble aggregates that lose tumor‑suppressive function. Meanwhile, these aberrant assemblies disrupt the function of molecular chaperones such as Hsp70 and Hsp90, which impairs p53 folding and further aggregation [146]. In parallel, aberrantly condensed and aggregated p53 exhibits less efficient clearance by the UPS and autophagy‐lysosome system. Defective protein folding, abnormal aggregation and impaired degradation jointly break protein homeostasis and attenuate the tumor‐suppressive capacity of p53 [147].

**FIGURE 4:**
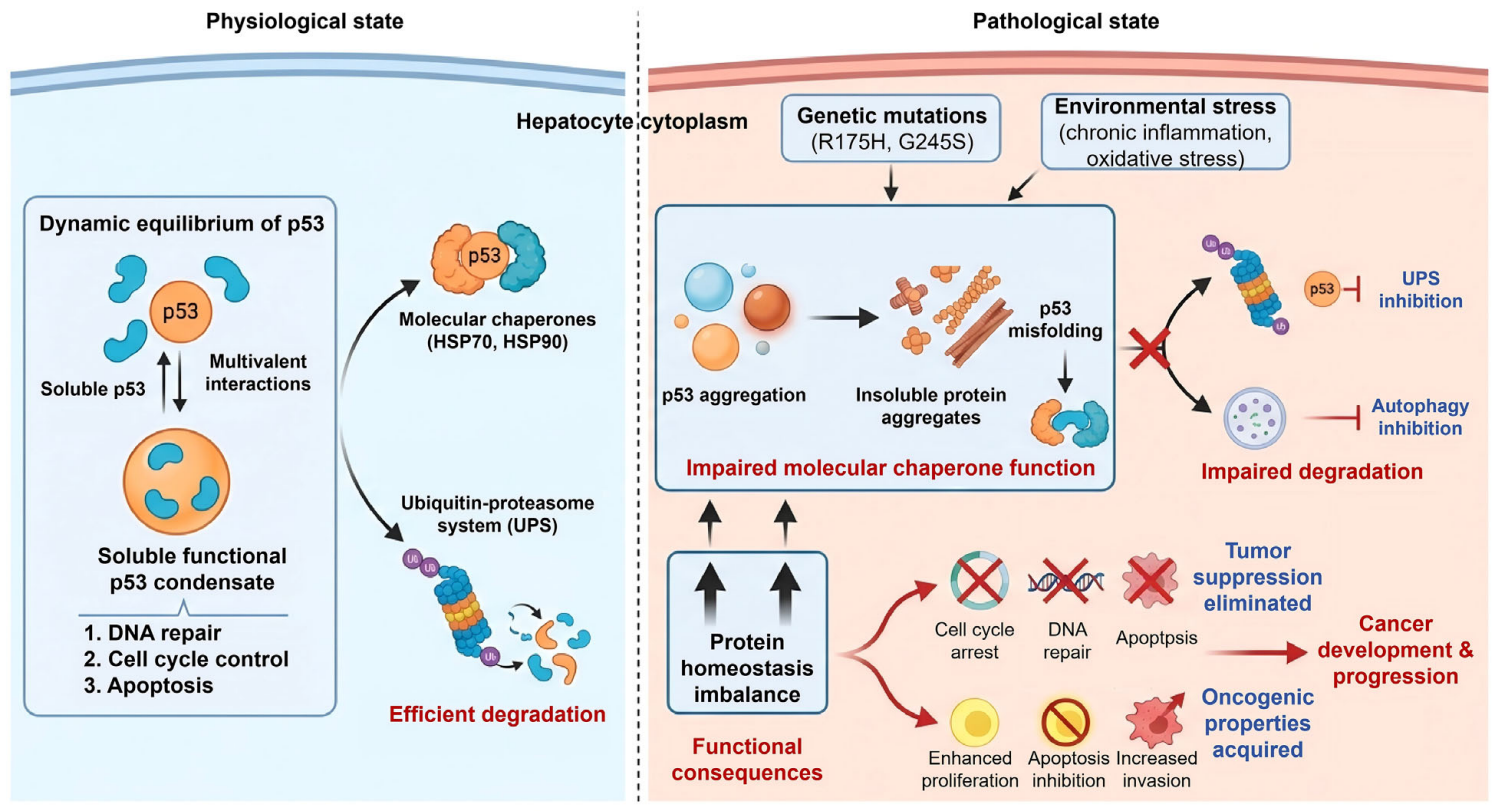
The schematic depicts that mutant p53 undergoes aberrant phase separation into insoluble aggregates, losing its tumor suppressor function.

### 3.2 Misregulated Gene Expression and Signal Transduction

Precise gene expression and signal transduction are fundamental for maintaining cellular homeostasis and adapting to environmental changes. Cells achieve spatiotemporally controlled gene activation through multilayered regulatory mechanisms and convert extracellular signals into intracellular responses [148]. BPS facilitates the assembly of transcription factors and signaling proteins into dynamic condensates, thereby enhancing transcriptional regulation and signal transduction efficiency. Aberrant phase separation triggers abnormal aggregation of such proteins within incorrect subcellular compartments [149]. This disturbs gene expression and impairs signaling cascades, ultimately fueling disease onset and progression.

#### 3.2.1 Aberrant Regulation of Gene Expression

BPS influences gene expression via mediating the assemble of transcription factors, orchestrating chromatin architecture, and facilitating RNA polymerase activation. Under physiological conditions, transcription factors can form dynamic condensates that coordinate with chromatin and transcriptional coactivators to regulate transcription [150]. Aberrant phase separation disrupts transcription factor assembly, impairs their interactions with chromatin and the transcriptional machinery, and leads to gene dysregulation [83].

Acute promyelocytic leukemia (APL) is a hematologic malignancy closely related to abnormal gene expression regulation. In patients with APL, the fusion transcription factor PML‐RARα undergoes abnormal phase separation or phase separation‐related assembly [151]. PML‐RARα is generated by a chromosomal translocation involving the promyelocytic leukemia gene (PML) and the retinoic acid receptor alpha gene (RARα) [152]. Under physiological conditions, RARα binds to retinoic acid and forms transcriptional complexes to modulate the expression of cell differentiation‐related genes. These transcriptional assemblies may rely on dynamic processes associated with biomolecular phase separation. However, the generation of PML‐RARα fusion protein alters its inherent assembly characteristics and triggers the formation of abnormally stable protein condensates or aggregates. These abnormal assemblies associate with target loci on chromatin and repress the expression of differentiation‐related genes, while more broadly dysregulating transcriptional programs associated with leukemogenesis [153, 154]. Dysregulated gene expression blocks the differentiation of myeloid progenitor cells. Immature leukemic cells thus accumulate in bone marrow and peripheral blood, disturbing normal hematopoiesis and eventually inducing the onset of APL.

#### 3.2.2 Aberrant Activation of Signal Transduction Pathways

BPS regulates signal transduction by modulating the assembly and interactions of signaling molecules. Under physiological conditions, signaling molecules dynamically assemble into complexes or condensates to facilitate signal transmission and amplification. Dysregulated phase separation remodels their assembly and distribution, leading to aberrant signal activation or suppression and disrupting cellular functions [155].

Aberrant signal transduction is a core driver of tumor progression. In colorectal cancer, the Wnt/β‐catenin pathway plays a particularly critical role [156]. Under normal conditions, β‐catenin is phosphorylated by the adenomatous polyposis coli (APC), glycogen synthase kinase‐3β (GSK‐3β) and other associated factors destruction complex and subsequently degraded via UPS [157]. However, upon Wnt stimulation, Wnt ligands bind to cell‐surface receptors and initiate downstream signal transduction to suppress the activity of the destruction complex. This process inhibits the phosphorylation and proteasomal degradation of β‐catenin, leading to its gradual accumulation in the cytoplasm. Subsequently, accumulated β‐catenin translocates into the nucleus and interacts with T‐cell factor/lymphoid enhancer factor (TCF/LEF) transcription factors to initiate the transcription of downstream target genes [158]. APC mutations compromise the function of the destruction complex and disrupt its dynamic organization via phase separation‐associated mechanisms. These defects block β‑catenin degradation, trigger persistent signaling activation, and further promote the initiation and progression of colorectal cancer.

### 3.3 Organelle Dysfunction‐Induced Diseases

Organelles such as mitochondria, endoplasmic reticulum, Golgi apparatus and lysosomes are subcellular structures that perform distinct physiological functions. BPS has emerged as an important regulator of organelle structure and function. Dysregulation of this process disrupts organelle homeostasis, which further facilitates the initiation and progression of diseases.

#### 3.3.1 Organelle Dysfunction

BPS and its associated condensates regulate numerous core biological processes within mitochondria including the arrangement of proteins related to mitochondrial dynamics, mitochondrial DNA (mtDNA) stability maintenance and the assembly of mitochondrial protein complexes. Aberrant phase separation may affect the regulation of mitochondrial fusion and fission, thereby contributing to mitochondrial morphological abnormalities, such as fragmentation or excessive fusion [159, 160]. Meanwhile, abnormal phase separation‐related assembly may interfere with mtDNA maintenance, replication, or transcription, thus compromising the expression of mitochondrial genes required for respiratory chain function. In addition, these abnormalities may disrupt the proper organization or assembly of mitochondrial respiratory chain complexes, resulting in impaired oxidative phosphorylation [161]. Mitochondrial dysfunction results in inadequate intracellular energy synthesis, frequently accompanied by excessive reactive oxygen species (ROS) production and aggravated oxidative stress [162]. These pathological alterations eventually trigger cellular injury and cell death, and are closely linked to numerous disorders such as Leber's hereditary optic neuropathy (LHON) and heart failure.

#### 3.3.2 Cellular Metabolic Reprogramming

Metabolic reprogramming is a common feature of tumor cells, enabling them to meet the energy demands and biosynthetic needs required for rapid proliferation. One of the best‐known examples is the Warburg effect, in which tumor cells preferentially rely on glycolysis for energy production even in the presence of oxygen, rather than primarily using oxidative phosphorylation [163, 164]. Emerging evidence suggests that BPS may participate in this metabolic shift. Glycolytic enzymes such as hexokinase (HK) and phosphofructokinase (PFK) are subject to BPS‐mediated regulatory control. Such regulatory mechanisms modulate the subcellular localization, intermolecular binding and catalytic activity of these enzymes [165]. Under physiological states, these enzymes assemble into dynamic biomolecular condensates. Such structures spatially compartmentalize glycolytic reactions in subcellular domains and precisely modulate metabolic flux. Aberrant phase separation disrupts the assembly characteristics of glycolytic enzymes and alters their subcellular localization, molecular interactions and catalytic efficiency [93]. These molecular abnormalities drive excessive or persistently activated glycolysis. This metabolic reprogramming furnishes energy and biosynthetic intermediates to sustain the rapid proliferation of tumor cells and facilitates tumor growth and malignant progression [166]. It also confers therapeutic resistance on cancer cells, which greatly raises the challenges of clinical tumor treatment.

### 3.4 Dysregulated Cellular Stress and Immune Responses

Cellular stress responses and immune responses are crucial defense mechanisms employed by cells and organisms to cope with external stimuli and pathogen invasion. BPS is involved in multiple steps of cellular stress responses and immune responses. When phase separation is aberrant, it results in abnormal cellular stress responses and dysregulated immune responses, thereby inducing diseases [100, 167].

#### 3.4.1 Aberrant Cellular Stress Responses

Cells initiate cellular stress responses upon exposure to extracellular stimuli including oxidative stress, heat shock and nutrient deprivation. Cells rely partially on the assembly of SGs to defend against cellular damage. SGs are dynamic biomolecular condensates formed by mRNAs and various proteins via BPS [168]. They can temporarily sequester mRNAs, suppress global translation, and at the same time protect mRNAs from degradation. Once the cellular stress is resolved, SGs rapidly disassemble, and the released mRNAs re‐enter translation, thereby restoring normal cellular functions.

Amyotrophic lateral sclerosis (ALS) is a fatal neurodegenerative disease closely associated with impaired cellular stress responses [169, 170]. In ALS, SG‐associated proteins such as TAR DNA‐binding protein 43 (TDP‐43) and fused in sarcoma (FUS) frequently harbor disease‐related mutations or undergo abnormal post‐translational modifications, leading to altered phase separation behavior [171]. This, in turn, promotes abnormal SG assembly, persistence, or stabilization. Aberrantly persistent SGs fail to disassemble normally after stress resolution, resulting in their accumulation within neurons. These accumulated SGs not only interfere with normal mRNA metabolism and protein translation but may also recruit additional aggregation‐prone proteins to form more stable or insoluble inclusions [30, 172]. These aberrant assemblies destroy the structural integrity and physiological function of neurons, eventually resulting in neuronal apoptosis. The progressive degeneration of motor neurons leads to clinical manifestations including muscle weakness, muscular atrophy and limb paralysis, and patients ultimately succumb to respiratory failure.

#### 3.4.2 Immune Responses Dysregulation

BPS governs the activation, proliferation and differentiation of immune cells as well as the synthesis and release of immune mediators, hence engaging in host immune defense responses. When phase separation becomes aberrant, it may impair immune cell function and alter immune signaling, leading to dysregulated immune responses and contributing to the development of autoimmune or infectious diseases [173].

Systemic lupus erythematosus (SLE) is a typical autoimmune disorder driven by disordered immune responses. Aberrant activation of the cyclic GMP‐AMP synthase‐stimulator of interferon genes (cGAS‐STING) signaling pathway acts as a key pathogenic factor in the development of this disease (Figure 5) [174]. The cGAS‐STING pathway is a central innate immune pathway through which cells sense aberrant cytoplasmic DNA, such as pathogen‐derived DNA or DNA released from damaged cells, and initiate immune responses. Under normal physiological conditions, when self‐DNA is abnormally released into cytoplasm, cGAS binds to DNA, and the cGAS‐DNA interaction can promote the formation of dynamic cGAS‐DNA condensates through phase separation‐related mechanisms [175, 176]. These condensates potentiate the catalytic activity of cGAS and facilitate its function in generating the second messenger cyclic GMP‐AMP (cGAMP). cGAMP subsequently binds to endoplasmic reticulum‐localized STING and triggers STING activation together with its trafficking toward the Golgi apparatus. Recruited signaling molecules including TANK‐binding kinase 1 (TBK1) and interferon regulatory factor 3 (IRF3) at the Golgi then assemble downstream signal complexes to initiate subsequent immune cascades. It ultimately promotes the generation of type I interferons (e.g., IFN‐β) and proinflammatory cytokines, maintaining innate immune response [177]. Defective clearance of self‐DNA results in cytoplasmic accumulation of self‐DNA in SLE patients. This accumulated DNA persistently activates cGAS and induces abnormal condensate assembly to maintain sustained cGAS hyperactivation. Meanwhile, aberrant post‐translational modifications of cGAS or STING alter their activation dynamics, resulting in persistent signal transduction and sustained production of type I interferons and proinflammatory cytokines. This disrupts immune tolerance and triggers autoimmune responses [178].

**FIGURE 5:**
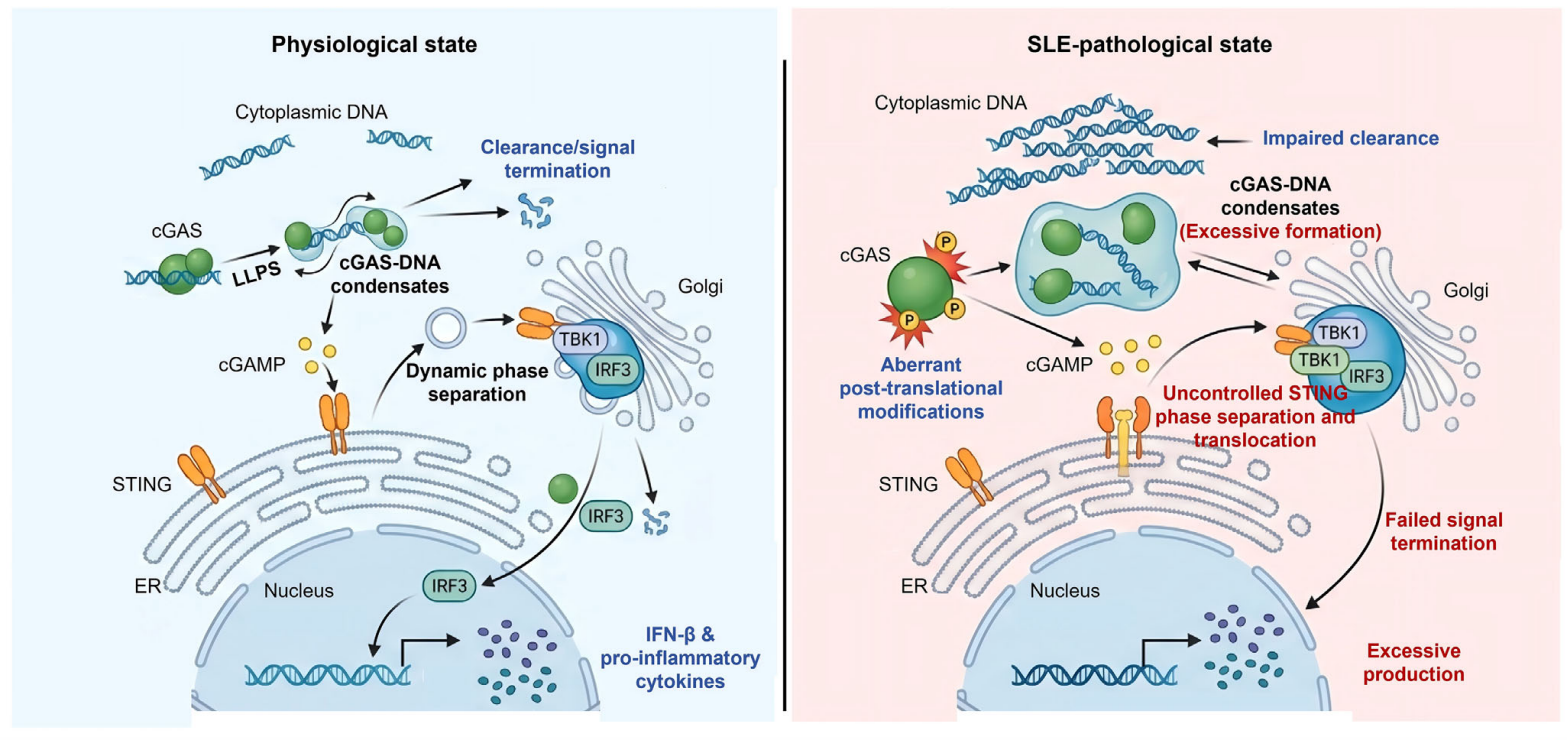
Abnormal phase separation of the cGAS‐STING pathway occurs in systemic lupus erythematosus.

Dysregulated BPS drives disease progression through interconnected pathological axes, from proteostasis failure to immune hyperactivation. A critical limitation of current research is its overreliance on correlative evidence: most studies link condensate abnormalities to disease phenotypes without establishing definitive causality. It remains unresolved whether altered phase behavior is a primary pathogenic driver or a secondary byproduct of cellular dysfunction. Furthermore, few findings have been validated in primary patient tissues, and condensate heterogeneity across disease subtypes is largely unexplored. Future studies should employ longitudinal clinical cohorts and targeted functional perturbations to distinguish causal pathological transitions from incidental changes, enabling the development of robust disease biomarkers.

## 4 BPS‐Targeted Drug Development

In recent years, BPS has gradually evolved from a biological concept into an actionable therapeutic target, emerging as a frontier in drug development. Traditional drugs primarily target protein with well‐defined structures. However, many disease‐related proteins lack stable conformations, rendering them “undruggable” targets [179, 180]. Elucidation of phase separation mechanisms offers new strategies to address this challenge. Small molecules, aptamers and peptides can be engineered to modulate phase separation and interfere with disease‐relevant biological processes. Such strategies broaden druggable targets and offer novel therapeutic approaches for intractable disorders. We classify BPS‐targeted therapeutic strategies into three mechanistically distinct modes: restoration of physiological condensate function, induction of therapeutic condensates, and modulation of pathological condensate maturation and material properties. This mechanism‐based framework captures the divergent molecular actions of individual interventions and offers a clearer conceptual foundation for rational therapeutic design.

### 4.1 Restoration of Physiological Condensate Function

This class of strategies corrects dysfunctional physiological condensates or enhances the assembly of native functional condensates, restoring their normal biological activities to counteract disease. They act on molecules that form condensates under healthy conditions but lose proper function in pathological states. Phase separation induction employs exogenous interventions (such as small‐molecule compounds, engineered peptides, or gene‐editing tools) to target disease‐associated molecules. This induces the formation of abnormal aggregates or accelerates the transition of physiological aggregates into toxic/dysfunctional states, ultimately disrupting key disease pathways and triggering cell death in diseased cells [181]. Such strategy is particularly suitable for diseases characterized by “high expression/high activity of target molecules”, typically including malignant tumors (overexpression of oncoproteins and abnormal activation of signaling pathways in tumor cells) and certain infectious diseases (massive replication of key pathogen proteins in host cells) [182, 183]. It can leverage the high abundance of target molecules in pathological cells to achieve “precision targeting”, minimizing interference with normal cells. Meanwhile, the induced BPS enables simultaneous blocking of multiple biological functions of target molecules (e.g., protein‐protein interactions, transcriptional regulation, and signal transduction), realizing “multipotent inhibition” [184]. In addition, it possesses unique intervention capabilities for traditional “undruggable” targets, expanding the scope of drug discovery targets.

As a key tumor suppressor, p53 maintains genomic stability by activating target genes related to DNA repair, cell cycle arrest, and apoptosis. Zong et al. found that alanyl‐tRNA synthetase (AARS1), acting as an intracellular lactate sensor and lactyl transferase, can form a lactate‐AMP intermediate in an ATP‐dependent manner [185] (Figure 6A). It transfers lactate to the K120 and K139 sites within the DNA‐binding domain of p53, impairing the LLPS and DNA‐binding ability of p53, thereby inhibiting its transcriptional activation function and tumor suppressor activity (Figure 6B,C). Through screening, it was discovered that β‐alanine, which has a molecular structure similar to that of lactate, can block the lactylation process by competitively binding to AARS1 and enhance the phase separation of p53. Beyond its effective regulation of tumors, BPS also exhibits unique advantages in the treatment of infectious diseases. Dai et al. demonstrated that AXIN1 functions as an efficient modulator of innate immune defense against DNA and RNA virus infections (Figure 6D) [186]. Under resting conditions, AXIN1 preserves IRF3 stability by inhibiting p62‐dependent autophagic degradation of IRF3. Following viral infection, phosphorylated TBK1 induces the phase separation of AXIN1 (Figure 6E). These condensates recruit IRF3 and TBK1 to facilitate IRF3 phosphorylation and subsequent type I interferon production, thus exerting antiviral effects against both DNA and RNA viruses (Figure 6F). However, the expression of AXIN1 is significantly suppressed in various viral infectious diseases. KYA1797K is a small‐molecule compound that specifically binds to the RGS domain of AXIN1. It enhances the binding affinity between AXIN1 and IRF3 (Figure 6G,H). This compound further facilitates the assembly and normal function of phase‐separated condensates, enabling AXIN1 to amplify antiviral immune responses relying on BPS.

**FIGURE 6:**
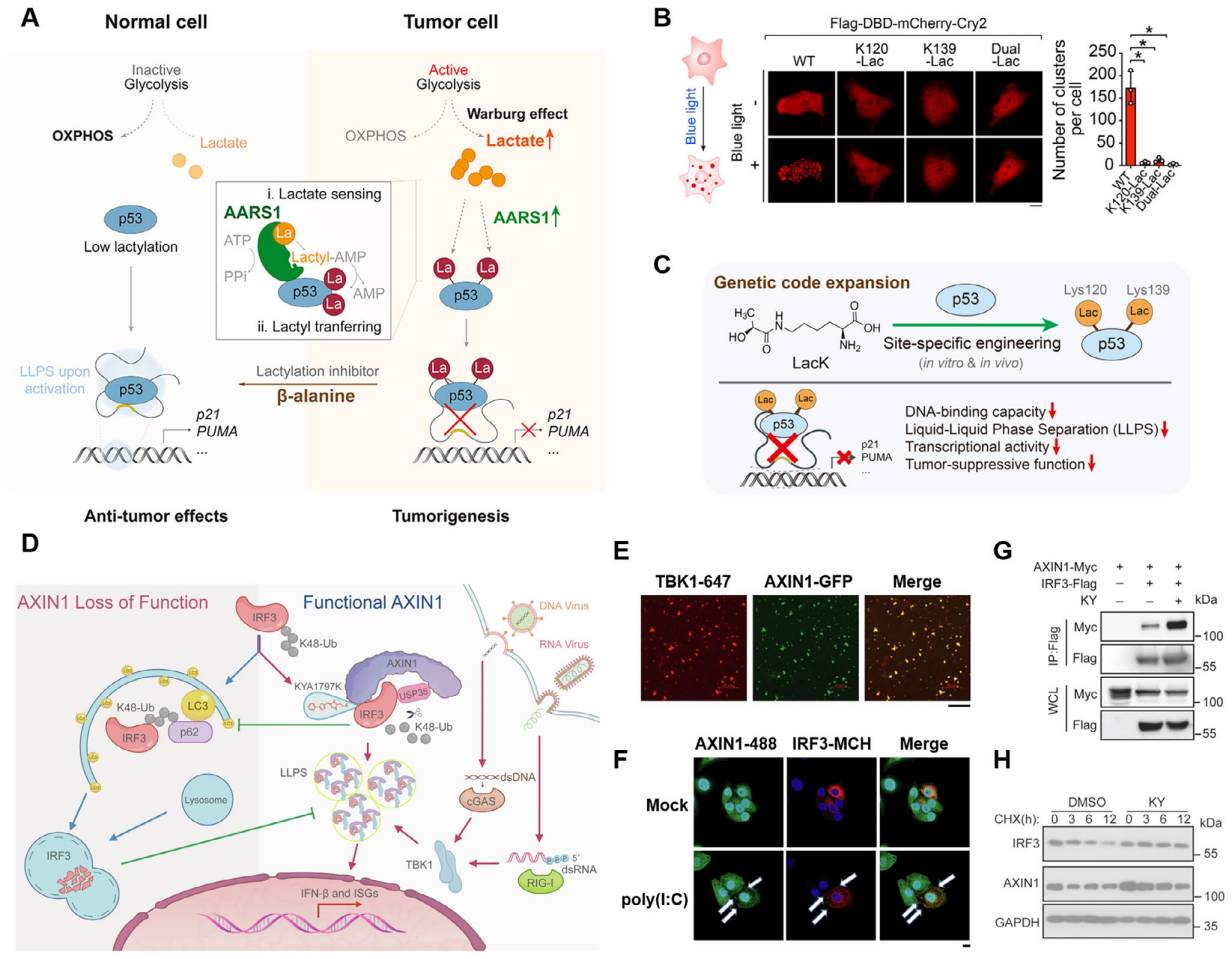
(A) AARS1 lactylates p53 at K120/K139, impairing p53 LLPS and DNA binding, which inhibits p53 transcriptional activity and tumor suppression. (B) Time‐lapse images of HEK293T cells expressing p53‐optoIDR constructs before and after light activation. (C) Summary of engineered system for site‐specific lactylation, demonstrating altered tumor‐suppressing capabilities of lactylated p53 [185]. Copyright 2024, Elsevier Inc. (D) Schematic model of AXIN1/IRF3‐governed type I IFN signaling. (E) TBK1‐mediated phosphorylation promotes AXIN1 LLPS and further recruits TBK1 into AXIN1 condensates. (F) Poly(I:C) stimulation induces robust cytoplasmic colocalization between AXIN1 condensates and punctate IRF3 assemblies [186]. Copyright 2024, Springer Nature. (G,H) KYA1797K strengthens the endogenous interaction between AXIN1 and IRF3, thereby extending IRF3 protein half‐life [187]. Copyright 2024, Springer Nature.

### 4.2 Induction of Therapeutic Condensates

These strategies trigger the formation of condensates that do not exist under physiological conditions. They leverage the sequestration, functional disruption or cytotoxic effects of newly assembled condensates to exert therapeutic effects, and are particularly suitable for eliminating pathogenic cells or suppressing deleterious molecular pathways. Nur77 acts as a pivotal molecule governing the survival and apoptosis of tumor cells. When localized in the nucleus, it promotes tumor growth. After translocating to mitochondria, it can interact with Bcl‐2 and convert it from an anti‐apoptotic molecule to a pro‐apoptotic molecule. To effectively regulate the Nur77/Bcl‐2 apoptotic pathway, Zhang et al. identified the compound XS561 that can directly bind to Nur77 through high‐throughput computer‐aided drug screening (Figure 7A) [177]. XS561 can specifically bind to the conformational regulatory domain of Nur77, induce a conformational change in Nur77‐LBD, expose the IDR at the N‐terminus to trigger phase separation, and trigger the nuclear‐to‐mitochondrial translocation of Nur77 (Figure 7B). Furthermore, it induces the phosphorylation of the LOOP domain of Bcl‐2 and the formation of droplets. Nur77 and Bcl‐2 eventually form condensates on mitochondria, triggering mitochondria‐dependent apoptosis in cancer cells. In addition to small‐molecule compounds, macromolecular therapeutics also leverage de novo condensate induction to achieve therapeutic outcomes. Tumor cells can form SGs to protect mRNAs and proteins, thereby promoting cell survival and leading to chemoresistance. Wang et al. designed an enzyme‐responsive phase‐separating peptide Y^SO4^F, the “sticker‐spacer‐sticker” structure can be converted into the phase‐separating peptide YF under the catalysis of lysosomal sulfatase, forming condensed droplets (Figure 7C) [187]. The FGDF domain in Y^SO4^F can target G3BP2, a core protein of stress granules, thereby fusing with SGs, blocking their sequestration of receptor for activated C kinase 1 (RACK1) protein, activating the MTK1‐p38‐caspase‐3 pro‐apoptotic pathway, and significantly enhancing chemotherapy efficacy. Transforming growth factor‐β1 (TGF‐β1) serves as a pivotal immunosuppressive mediator during tumor initiation and progression, primarily exerting biological functions by shaping an immunosuppressive tumor microenvironment and facilitating tumor immune evasion. Xing et al. found that cationic polymers (PEI, cDex, DETA‐Dex) possess potent RNA phase separation‐inducing activity (Figure 7D) [188]. They can specifically encapsulate and degrade TGF β1 mRNA by forming RNA droplets, reducing the immunosuppressive capacity of tumor cells and activating anti‐tumor immune responses (Figure 7E). To address the issues that traditional single‐target antibiotics are prone to developing resistance due to bacterial mutations (e.g., point mutations in target proteins, enhanced efflux pumps), and that traditional multi‐target strategies face problems such as pharmacokinetic inconsistencies and cytotoxicity, Yang et al. proposed a bacterial intracellular order disruption strategy. By designing diarginine‐mimetic peptides (dAPMs), the authors used bacterial DNA as an intracellular trigger to induce ligand–receptor interaction‐mediated phase separation (Figure 7F) [189]. The resulting intracellular condensates showed broad binding affinity for bacterial proteins and markedly disrupted essential physiological processes. They also damaged bacterial membranes through an inside‐out mechanism, thereby exerting potent antibacterial activity and limiting the development of drug resistance.

**FIGURE 7:**
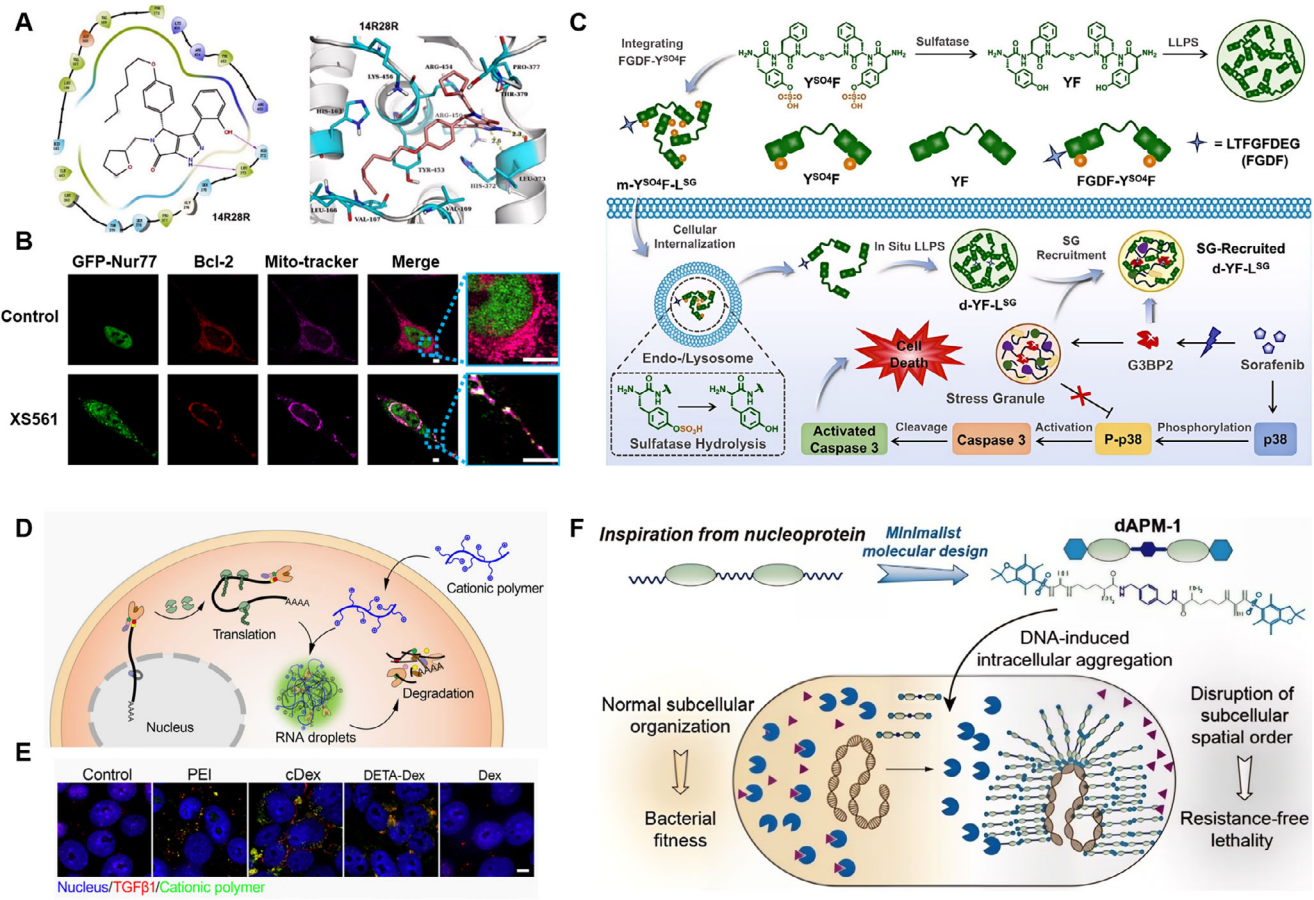
(A) Molecular docking simulation of XS561 binding to the Nur77 ligand‐binding domain identifies XS561_14R28R as the core bioactive component responsible for Nur77 binding. (B) Confocal imaging verified that XS561 treatment triggers the formation of GFP‐Nur77/Bcl‐2 condensates at the mitochondrial membrane [177]. Copyright 2024, Elsevier B.V. (C) Schematic illustration of intracellular in situ peptide phase separation for SG‐targeted coacervate formation, enabling sorafenib‐based cancer chemotherapy [187]. Copyright 2025, Wiley‐VCH GmbH. (D) Intracellular cationic polymers undergo LLPS with endogenous RNA to form RNA condensates. (E) Fluorescence images of the colocalization of TGF‐β1 mRNA with cationic polymers in 4T1 cells. Copyright 2022, Springer Nature. (F) Intracellular DNA triggers the aggregation of dAPMs via ligand‐receptor interactions [189]. Copyright 2024, American Chemical Society.

### 4.3 Modulation of Pathological Condensate Maturation and Material Properties

This category of therapeutics targets pathologically aberrant condensates to normalize their material and functional properties. Rather than introducing new assemblies, these interventions restore cellular homeostasis by modulating condensate assembly dynamics, reshaping their maturation trajectory and reversing aberrant hardening. Its mechanism involves interfering with the interactions of key phase separation molecules (e.g., hydrophobic interactions, electrostatic interactions, multivalent binding) or regulating phase separation‐related regulatory factors (e.g., RNA, post‐translational modification enzymes) to inhibit disease‐associated aberrant phase separation processes (e.g., formation of pathological condensates, excessive assembly of physiological condensates) and restore intracellular phase separation homeostasis [136]. This strategy is mainly applicable to diseases where aberrant phase separation serves as the core pathological driver (including neurodegenerative diseases, tumors, metabolic diseases, etc.). In the occurrence and progression of breast cancer, Forkhead box protein M1 (FOXM1) serves as a key phase‐separating oncoprotein. This protein undergoes LLPS mediated by IDR1, binds to FKH consensus DNA sequences, and sequesters transcriptional apparatus in the cell nucleus [190]. Such regulatory behavior preserves chromatin accessibility and stabilizes the super‐enhancer landscape required to sustain metastatic progression of tumor cells, thereby continuously activating the transcription of downstream oncogenic target genes and promoting the proliferation, migration, and invasion of tumor cells. Xie et al. identified that AMP‐activated protein kinase (AMPK) agonists can inhibit the phase separation of FOXM1 by phosphorylating the S376 site within its IDR (Figure 8A) [191]. This impairs the binding ability of FOXM1 to the DNA elements of its target genes, consequently suppressing the expression of downstream pro‐oncogenes (Figure 8B). Furthermore, they designed the interfering peptide FIP4, which targets IDR and contains the AMPK‐phosphorylated residue (Figure 8C). The peptide significantly inhibits the expression of pro‐oncogenes and enhances tumor immunogenicity. Tumor cell‐expressed programmed death‐ligand 1 (PD‐L1) is one of the major inhibitory factors of anti‐tumor immune surveillance. However, the population benefiting from PD‐1/PD‐L1 axis immunotherapy remains limited. Hu et al. were the first to discover that under interferon‐γ (IFNγ) stimulation, the histone acetyltransferase KAT8 undergoes phase separation with the transcription factor IRF1 through multivalent interactions (Figure 8D) [192]. The formed KAT8‐IRF1 condensates can promote the acetylation of IRF1 at the K78 site, enhance its binding ability to the PD‐L1 promoter, and recruit the transcriptional machinery to facilitate the transcription of PD‐L1 mRNA. Based on the mechanism, they designed the inhibitory peptide 2142‐R8. This peptide interferes with the assembly of KAT8‐IRF1 condensates, thereby downregulating PD‐L1 expression and amplifying antitumor immune responses (Figure 8E). Androgen deprivation therapy (ADT) remains the principal treatment modality against castration‐resistant prostate cancer (CRPC). Traditional anti‐androgens inhibit androgen receptor (AR)‐mediated transcriptional activation by targeting the ligand‐binding domain (Figure 8F) [193]. Nevertheless, anti‐androgens can promote the phase separation of drug‐resistant mutant AR in a ligand‐independent manner, which in turn restores AR transcriptional activity and leads to the continuous proliferation of cancer cells (Figure 8G). Through phenotypic screening targeting LLPS, Xie et al. identified the small‐molecule compound ET516 (Figure 8H,I) [194]. It directly binds to the intrinsically disordered N‐terminal domain (NTD) of AR to specifically disrupt AR condensates, thereby effectively inhibiting the transcriptional activity of drug‐resistant mutant AR and exhibiting significant inhibitory activity against prostate cancer cells harboring drug‐resistant mutant AR.

**FIGURE 8:**
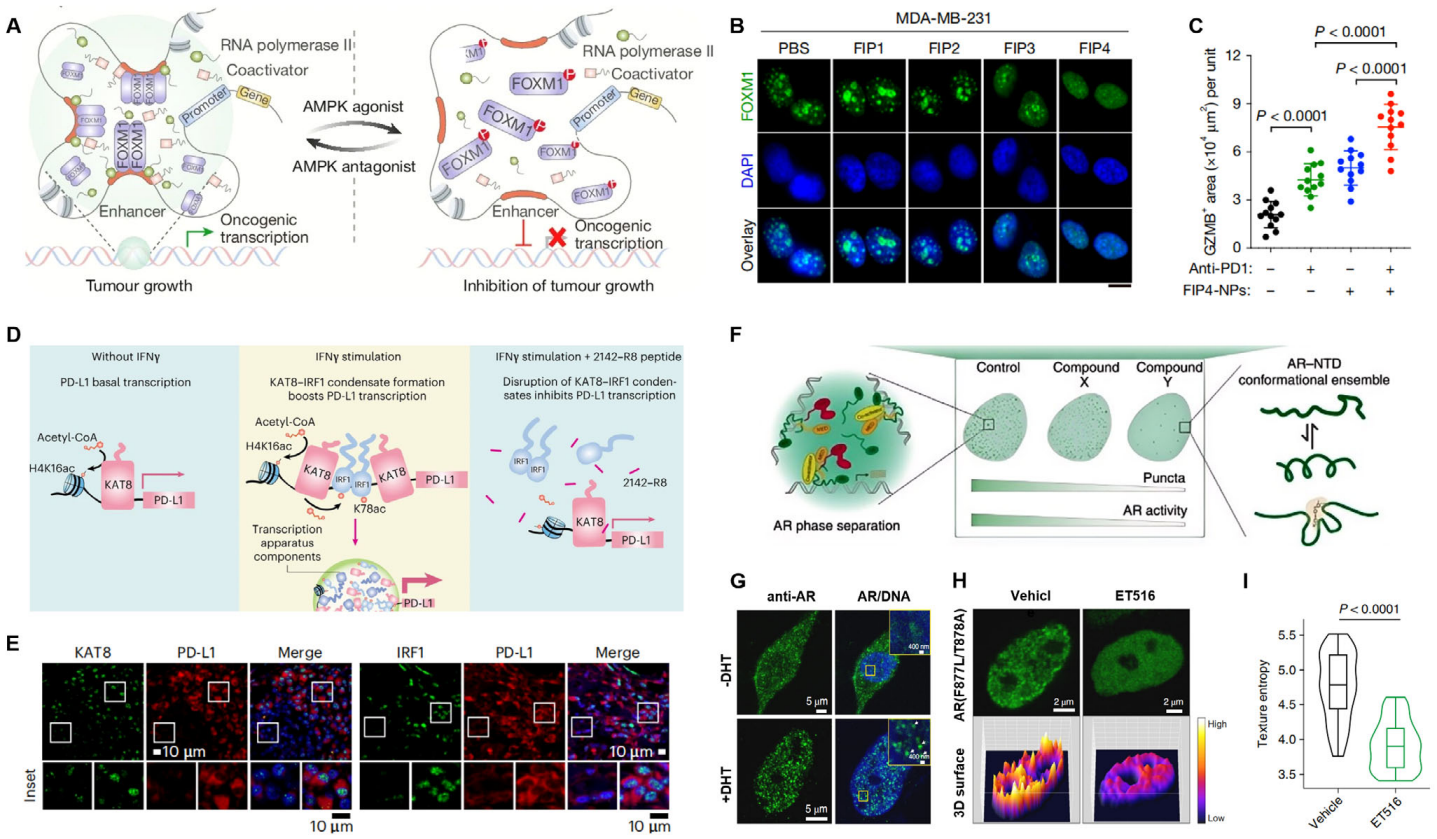
(A) Schematic depicting AMPK‐governed FOXM1 phase separation and transcriptional regulation [191]. (B) Cellular assays verified that FIP4 exerts a more robust suppressive effect on FOXM1 condensation than FIP3. (C) Combination therapy with FIP4 and anti‐PD‐1 increases intratumoral CD8+ T cell infiltration and granzyme B release. Copyright 2025, The Author(s), under exclusive licence to Springer Nature Limited. (D) KAT8 undergoes co‐phase separation with IRF1, which promotes IRF1 acetylation at K78. (E) Co‐immunofluorescence staining of clinical lung cancer tissues for PD‐L1 with KAT8 or IRF1 [192]. Copyright 2026, Wiley‐VCH GmbH. (F) Ligand‐activated AR undergoes LLPS through its intrinsically disordered N‐terminal domain to form transcriptionally active condensates. (G) Dihydrotestosterone stimulation promotes uniform nuclear translocation of endogenous AR in prostate cancer cells, whereas ligand‐free AR is evenly distributed in the cytoplasm [193]. Copyright 2024, American Association for Cancer Research. (H,I) Tumor tissue imaging demonstrated that ET516 effectively blocks AR LLPS, remarkably reducing punctate condensate formation in xenograft tumor tissues [194]. Copyright 2022, The Author(s), under exclusive licence to Springer Nature America, Inc.

In addition to its significant advantages in regulating tumor proliferation, drug resistance, and immunosuppression, inhibiting aberrant phase separation also demonstrates clear value in the treatment of neurodegenerative diseases (NDs). TDP‐43 is a key pathogenic protein in NDs. The conserved region (CR) within the amyloid core of its low‐complexity domain (LCD) undergoes a conformational transition from a physiological α‐helical structure to β‐sheet‐rich assemblies, triggering aberrant phase separation and aggregation of TDP‐43 to form solid amyloid aggregates (Figure 9A) [195]. Meanwhile, TDP‐43 undergoes phosphorylation at serine residues 409/410 (pTDP‐43). These phosphorylated aggregates not only lose RNA‐binding ability but also interfere with RNA metabolism, further promoting disease progression. Sun et al. proposed an AI‐based de novo protein binder design strategy. The identified inhibitor B9 exhibits extremely high binding affinity (87.9 ± 2.7 nM) for the CR region of TDP‐43 (Figure 9B) [196]. It stabilizes the α‐helical conformation through hydrophobic interactions, directly blocking the pathological transition to β‐sheets, inhibiting aggregation at the source, and significantly reducing the level of the disease‐specific biomarker pTDP‐43 (S409/410) (Figure 9C). Zhang et al. propose that nuclear TDP‐43 is an important regulator of gene expression, and TDP‐43 aggregation (TDP‐43 nuclear bodies, NBs) also occurs in the nucleus, whose solidification leads to functional abnormalities [197]. Traditional drugs cannot effectively penetrate the cell nucleus and can only inhibit TDP‐43 phase separation in the cytoplasm, failing to address the pathological issue of nuclear TDP‐43 aggregation. To this end, they designed and synthesized halogen‐doped graphene quantum dots (GQDs‐Cl, Cl‐GQDs‐OH) (Figure 9D). With improved nuclear permeability, these quantum dots directly interact with multiple domains of TDP‐43 and modulate its phase separation via hydrophobic and electrostatic forces. Under oxidative stress or hyperosmotic conditions, they effectively inhibit TDP‐43 nuclear bodies assemble and SGs, reducing their amyloid aggregation and disease‐associated phosphorylation at S409/410 (Figure 9E,F). Beyond TDP‐43, tau protein is closely associated with various neurodegenerative diseases. Under normal physiological conditions, tau protein functions to bind microtubules and regulate their assembly into bundles. Pathologically modified tau protein drives phase separation, ultimately forming microtubule‐binding‐region‐based insoluble fibrils and neurofibrillary tangles. Huang et al. found that methylene blue (MB) can bind to the entire region of tau protein through extensive hydrophobic and electrostatic interactions, significantly promoting tau LLPS and further accelerating the transition of tau droplets to a gel‐like state (LGPS) (Figure 9G) [198]. Conformational analysis revealed that MB binding induces tau to unfold from its native “paperclip‐like” compact structure. Although this unfolded conformation facilitates LLPS and gelation, it blocks the transition to toxic fibrillary aggregates caused by LSPS, ultimately reducing the toxicity of tau aggregates to neural cells.

**FIGURE 9:**
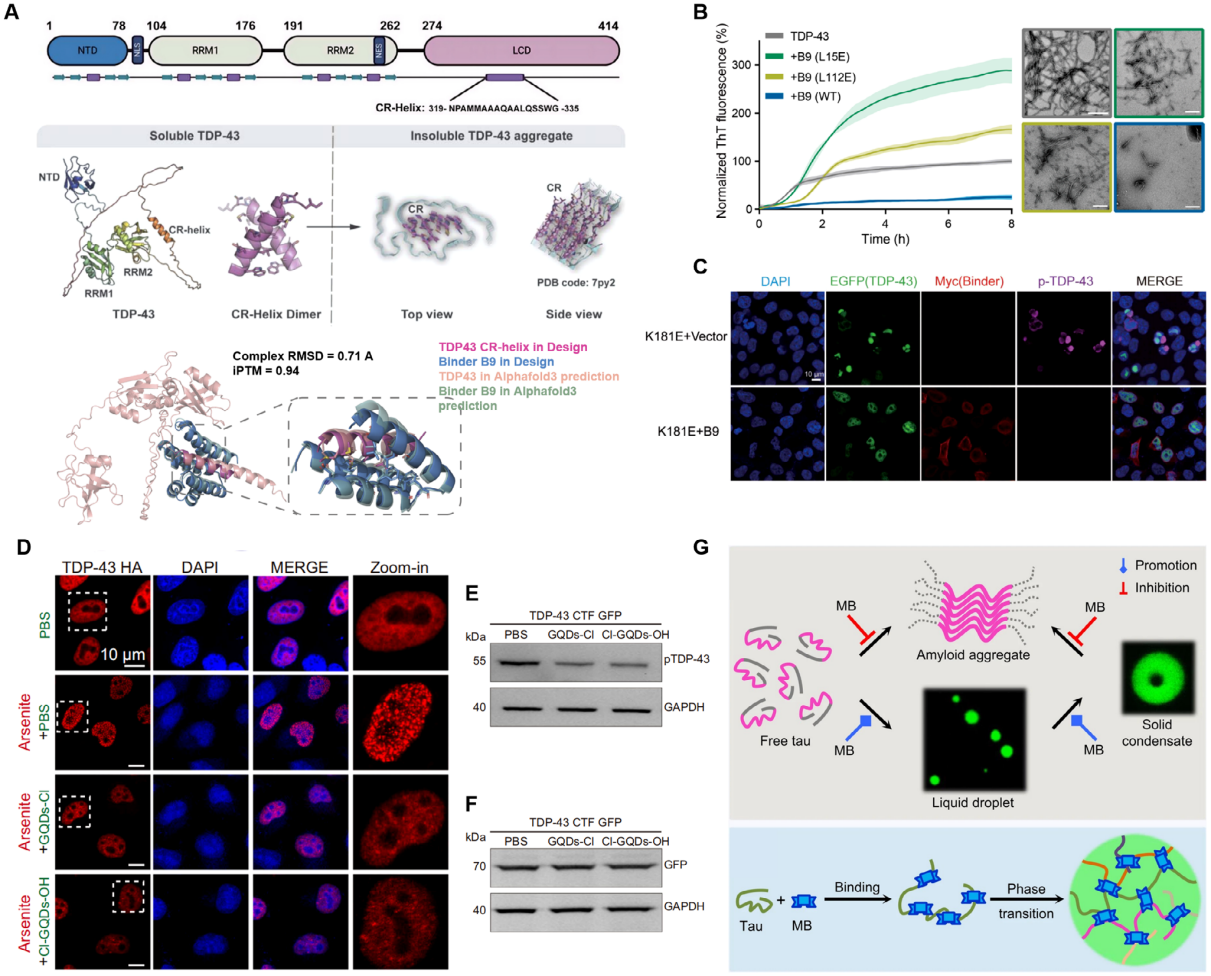
(A) Schematic of TDP‐43 functional domains, predicted secondary structures, and its structural transition from soluble monomers to pathological aggregates. (B) ThT staining and negative‐stain TEM image. (C) Representative fluorescence images of HEK293T cells co‐expressing TDP‐43 K181E and B9 [195]. Copyright 2025, the Author(s). (D) Confocal images of TDP‐43‐transfected HeLa cells. (E,F) Western blot assays for phosphorylated TDP‐43 (S409/410) and GFP in transfected 293T cells [197]. Copyright 2024, Springer Nature. (G) MB suppresses free tau aggregation and blocks the maturation of tau gel droplets into insoluble aggregates [198]. Copyright 2023, Springer Nature.

Therapeutic strategies that either induce or inhibit phase separation have delivered encouraging preclinical results in cancer and neurodegenerative disease models. However, nearly all currently available modulators fail to distinguish pathological condensates from their physiological counterparts, creating significant off‐target toxicity risks. The field also faces bottlenecks in subcellular drug delivery and standardized in vivo efficacy assessment, with most candidates validated exclusively in cultured cell lines. To advance toward clinical translation, the community must develop high‐throughput selectivity screening platforms and non‐invasive in vivo imaging tools that enable real‐time monitoring of target engagement.

## 5 BPS‐Inspired Biomaterial Design

Under physiological conditions, BPS is driven by multivalent weak interactions, such as π–π stacking, hydrogen bonding, and electrostatic attraction. When concentrations exceed the critical threshold, intrinsically disordered regions and multivalent folded domains drive the spontaneous formation of condensates. This process precisely regulates cellular processes including reaction kinetics, signal transduction, and transcription, while maintaining gene expression, stress resistance, and metabolic homeostasis [58, 199]. These condensates are dynamically reversible, responding to environmental changes through bidirectional assembly and disassembly. They enhance local molecular concentrations and reaction efficiency via selective enrichment, and prevent side reactions through spatial segregation [200]. Moreover, their membrane‐less nature endows them with mechanical properties compatible with soft tissues and adaptable morphologies ranging from spherical to tubular or fibrous [201, 202]. These unique properties and structural features of phase separation‐driven condensates offer important insights for the design of novel biomaterials. Recent designs of intrinsically disordered region‐mimetic peptides enable programmable coacervation with tunable cargo selectivity [203]. Meanwhile, the condensates crosslinked by dynamic covalent bonds permit externally triggered cargo release [204]. The above information illustrates that the physical principles of phase separation are guiding the engineering design and development of next‐generation biomaterials.

### 5.1 General Design Principles of BPS‐Based Biomaterials

BPS‐inspired biomaterials borrow physicochemical features from native liquid‐liquid, liquid‐gel and liquid‐solid condensates. They operate under the property‐design‐function paradigm. Molecular‐scale engineering reshapes condensate properties to realize target biomedical functions. Key adjustable material traits cover phase state, assembly‐disassembly reversibility, multivalent enrichment capability, environmental response threshold, mechanical modulus and biocompatibility. Researchers tune phase transitions via multivalent non‐covalent interactions. IDR‐mimicking sticker‐spacer peptides or nucleic acids produce LLPS liquid assemblies. These assemblies retain high molecular diffusivity and dynamic fusion‐fission behaviors for protocell simulation, intracellular delivery and signal‐amplified imaging. Moderate dynamic cross‐linking drives condensates into the LGPS gel state to build mechanically stable tissue‐engineering scaffolds. Uncontrolled molecular stacking triggers harmful LSPS solidification. Delivery‐oriented materials must prevent such irreversible, cytotoxic aggregation. Stimuli‐responsive molecular switches offer spatiotemporal control over condensate assembly and disassembly. Endogenous cues including pH shifts, ionic‐strength changes, protease cleavage and bio‑orthogonal click reactions trigger in‑situ condensation at disease sites and cut down off‑target risks. Exogenous inputs such as light and temperature enable remote behavior modulation. Adjusting the ratio of charged and hydrophobic building blocks optimizes response thresholds for pathological microenvironments.

Multivalent enrichment, an innate feature of phase‑separated assemblies, serves as another core design pillar. Multivalent backbones made of peptides, DNA or cationic polymers integrate selective‑binding moieties. These moieties capture therapeutic cargoes or signal‑related molecules. Engineers balance binding affinity carefully. Sufficient affinity ensures satisfactory loading capacity. Moderate interaction strength allows stimulus‑triggered cargo release and avoids toxic permanent molecular sequestration. Biocompatibility and hierarchical structural matching are essential for real‑world biological deployment. Biogenic building blocks like gelatin, histones, short peptides and nucleic acids are preferred. They reduce immunogenicity and cytotoxicity. Material mechanics match practical application scenarios. Liquid condensates fit intracellular delivery. Gel‑phase networks adapt to soft‑tissue regeneration. Solid‑state assemblies fulfil barrier‑mimicking demands. Nanoscale coacervate droplets can assemble into micro‑vesicles or macro‑porous 3D‑printable constructs that recapitulate extracellular matrix features. Embedded degradable motifs enable programmable in vivo degradation aligned with tissue‑repair progress. Combined, these interrelated rules execute the property‑design‑function workflow. Modular adjustments to phase behavior, responsive switches, multivalent enrichment and biocompatibility deliver reliable biomaterial performance.

### 5.2 Protocell Systems Mimicking Native Cellular Behaviors

Constructing materials for simulating primordial cell behaviors is a key research direction at the intersection of modern life sciences and materials science, which can provide crucial clues for understanding the evolutionary origins of complex cellular functions [205]. In vitro construction of cell‐mimetic materials enables the exploration of pathological mechanisms arising from aberrant cellular functional evolution at the single‐cell level, which further accelerates the development of targeted therapeutic strategies. Meanwhile, recapitulating the core structures and functions of cells helps overcome the limitations of traditional artificial materials, such as single function and delayed response [206, 207]. The construction of traditional primordial cell models mostly relies on lipid bilayer vesicles or polymer microspheres. Although these models can simulate compartmentalized structures, they have significant limitations in functional simulation. The membrane structure of liposomes is fragile and prone to damage under extreme environments (e.g., high ionic strength, proteases), and it is difficult to achieve efficient enrichment of macromolecules such as enzymes and nucleic acids as well as the sustained progression of metabolic reactions [208, 209]. Although polymer microspheres have improved stability, their rigid structure cannot dynamically respond to environmental changes, and their poor biocompatibility makes it difficult to simulate the dynamic life characteristics of primitive life. In contrast, BPS materials offer a novel approach to overcoming these limitations.

In eukaryotic cells, it is well known that membraneless subcellular structures formed through phase separation exhibit liquid‐like morphologies and viscoelastic properties. However, the fundamental question of how molecules are transported inside these highly crowded and structurally complex biomolecular condensates remains unresolved. For a long time, the scientific community has generally believed that the mass exchange and molecular diffusion in condensates comply with the classical Fick's Law of Diffusion [210]. Which dictates that the diffusion rate increases with the square root of time, accompanied by a diffuse diffusion front and a gradually weakened concentration gradient. Nevertheless, this model fails to account for the sharp diffusion boundaries and rapid transport observed in certain experiments. Andreas Walther constructed a DNA phase separation model system with two functionalized long single‐stranded DNA (ssDNA) copolymers as the core components (Figure 10A) [211]. Based on the specific binding between invading molecules (m*) and m barcodes (molecular recognition) within the condensates, they observed a brand‐new “Ballistic Wave Diffusion” diffusion behavior (Figure 10B). This diffusion mode is characterized by two distinct features: the highly sharp front and the linear propagation velocity (ΔX ∝ t) (Figure 10C,D). It subverts the scope of application of traditional diffusion theories in condensates and provides a theoretical basis for the design of artificial condensate systems and the intervention of related diseases (such as abnormal condensates in neurodegenerative diseases). Beyond deciphering cellular behaviors, mimicking the dynamic processes of biomimetic cells facilitates the development of active materials with life‐like characteristics. Although several in vitro active systems exist, such as actin‐myosin and microtubule‐kinesin, along with studies combining artificial molecular machines with liquid crystals and gels. While, current systems still lack spatiotemporal control over collective motion, making it difficult to simulate the precise dynamic behaviors of cells. Zhao et al. designed a light‐responsive active fluid based on Y‐shaped DNA nanostructures (Y‐motifs) [212]. Leveraging the azobenzene‐based photoswitches at the sticky ends of Y‐motifs, this fluid can achieve nanoscale mechanical motions under the spatiotemporal regulation of visible light (Vis) and ultraviolet light (UV) (Figure 10E). These nanoscale motions can be amplified into macroscopic cell‐like behaviors, including amoeba‐like movement, controllable deformation, fission, and rotation (Figure 10F). This study not only offers novel insights into the development of intelligent materials with life‐like characteristics but also provides a new perspective for understanding the physical principles underlying coordinated motion in active substances.

**FIGURE 10:**
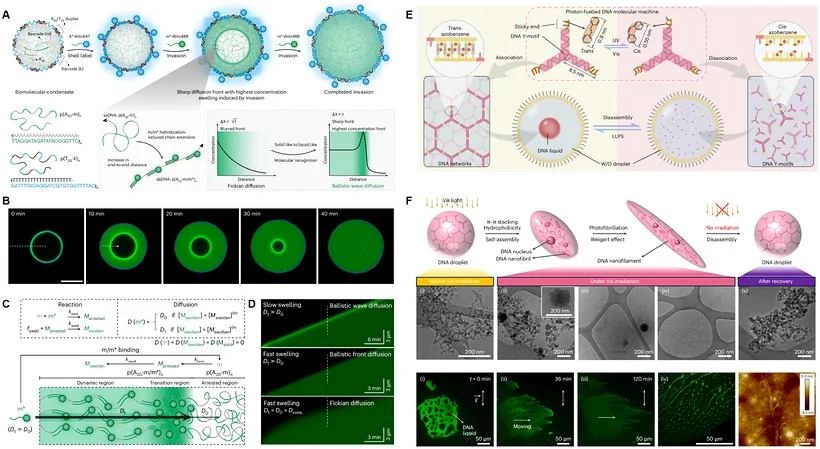
(A) Schematic overview of ballistic wave diffusion in DNA condensates. (B) Time‐lapse confocal microscopy images visualize the dynamic process of exogenous molecules invading DNA condensates. (C) Fundamental chemical reaction and diffusion mechanisms underlying the ballistic wave diffusion model [211]. Copyright 2025, Springer Nature. (D) Typical simulated kymograph profiles intuitively illustrate the features of ballistic wave diffusion behavior. (E) Design of azobenzene‐functionalized Y‐shaped DNA nanodevices as nano‐energy transducers for photocontrollable reversible assembly of DNA condensates. (F) Schematic diagrams and cryo‐TEM images illustrate the dynamic photoresponsive structural evolution of DNA systems [212]. Copyright 2025, The Author(s), under exclusive license to Springer Nature Limited.

### 5.3 Active Drug Delivery Platforms

BPS provides a highly promising strategy for constructing drug delivery platforms. These condensates, formed through BPS, exhibit high efficiency in drug loading and transmembrane delivery while effectively maintaining the biological activity of the loaded drugs. Inspired by natural vesicular compartments, Wen et al. described a LLPS‐based vesicle assembly using nucleic acid‐protein complexes (Figure 11A) [213]. Specifically, cholesterol‐conjugated single‐stranded DNA and histones self‐assemble at designated component ratios (Figure 11B,C). By regulating the ratio and concentration of the two components, condensed vesicles (CVs) characterized by low‐density cavities are formed. This layered architecture, characterized by a high‐density liquid outer shell and a low‐density aqueous inner lumen, exhibits exceptional structural stability (Figure 11D). It significantly enhances drug loading efficiency while preserving the activity of the loaded biological drugs (such as viral particles, mRNA, cytokines, etc.). To achieve the controllable preparation of drug delivery condensates with tailored sizes, Sun et al. developed stable nanoscale condensates based on histidine‐rich beak protein peptides (HBpep) through PEGylation modification and adjusting the ratio of PEGylated to non‐PEGylated peptides (Figure 11E) [214]. Precise control over the size and stability of peptide condensates (CMs) effectively overcomes key limitations of conventional CMs, including their tendency to fuse and their unsuitable dimensions for biological applications (Figure 11F,G). In addition to favorable biological stability and high delivery efficiency, the dynamic tunability, environmental responsiveness, and concentration‐dependent assembly behavior of condensates collectively confer unique advantages on BPS‐based drug delivery platforms, offering a novel design strategy for precision medicine (Figure 11H,I).

**FIGURE 11:**
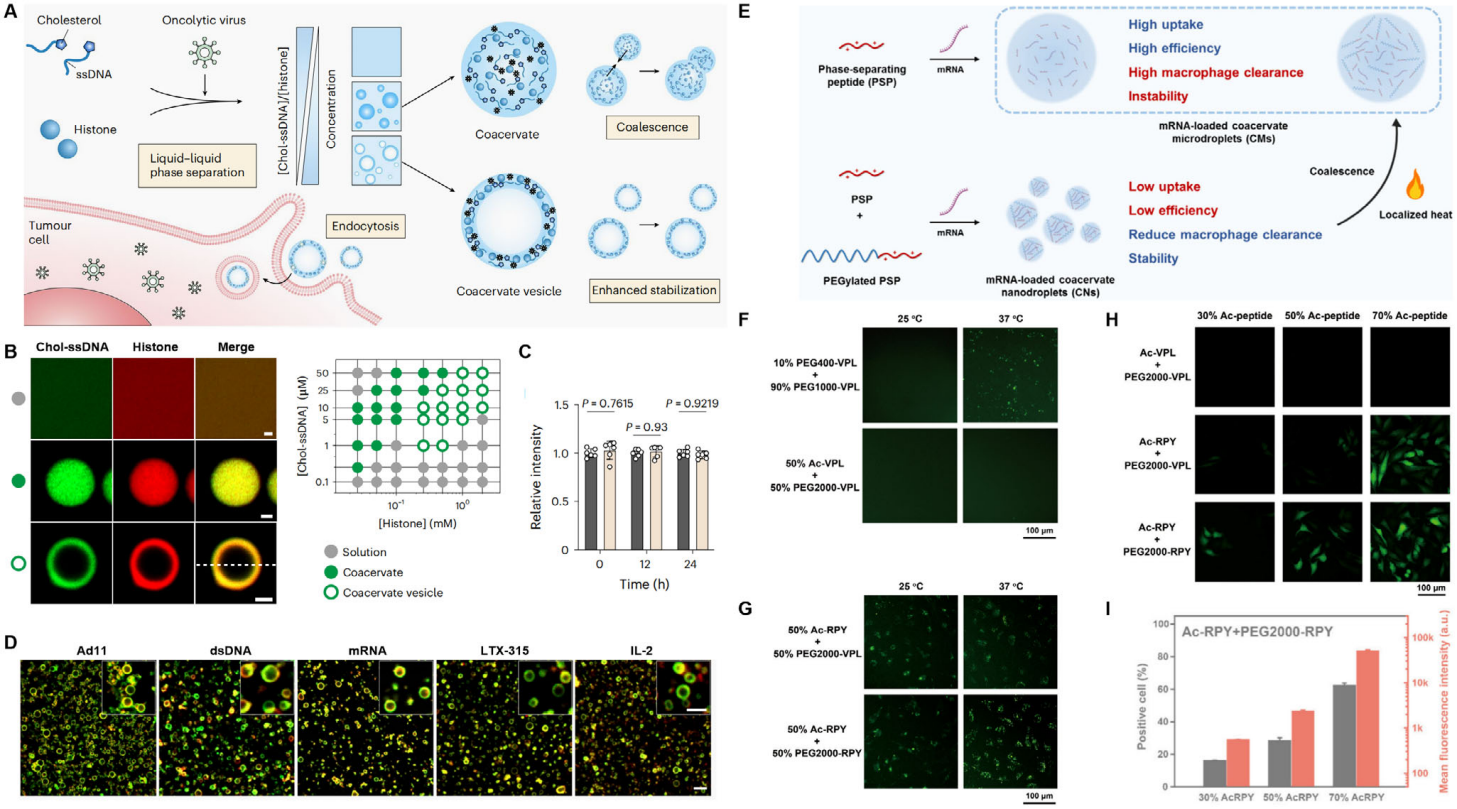
(A) Schematic illustration of the construction of coacervate vesicles for biotherapeutic delivery. (B) Mixtures of histone‐ and cholesterol‐modified single‐stranded DNA exhibit tunable phase behavior at different component ratios and concentrations. (C) Detection of GFP expression in HEK‐293 cells treated with free ad11 or CV‐released ad11. (D) Fluorescence colocalization analysis of 6‐FAM‐modified Chol‐ssDNA and Cy5‐labeled bio‐cargos [213]. Copyright 2025, The Author(s), under exclusive licence to Springer Nature Limited. (E) Schematic diagram of thermosensitive coacervate nanodroplets (CNs). (F,G) Fluorescence imaging of HeLa cells incubated with peptide‐based CNs at 25°C and 37°C. (H,I) Fluorescence detection of EGFP mRNA‐transfected HeLa cells via peptide CN delivery systems [214]. Copyright 2024, Springer Nature.

#### 5.3.1 Stimulus‐Triggered Controlled Drug Release

In biological systems, the formation of BPS is co‐regulated by intrinsic and extrinsic factors. Intrinsic factors mainly include protein concentration and structural characteristics, while extrinsic factors encompass temperature, pH value, salt ions, ATP, and other molecules. Such dynamic tunability and environmental responsiveness provide insights into the construction of drug delivery platforms [215]. Enabling precise drug release in response to the physiological environment or external stimuli can enhance therapeutic efficacy and reduce side effects.

Protein‐based drugs (e.g., antibodies, enzymes, cytokines) offer advantages of high specificity and low side effects, but their inability to independently cross cell membranes to exert effects in target cells has become a critical bottleneck for clinical applications. Although existing delivery carriers can mediate transmembrane transport of proteins, they often fail to efficiently enter the cytoplasm to perform their functions [216]. BPS commonly observed in natural systems, enables efficient enrichment and protection of macromolecules, thus being regarded as a potential source of inspiration for drug delivery. Bao et al. reported a photo‐responsive phase‐separating fluorescent molecule (PPFM) (Figure 12A) [217]. This molecule can condense into fluorescent droplets via LLPS in aqueous solutions and revert to a monomeric state upon 405 nm laser irradiation, thereby achieving depolymerization (Figure 12B). Based on multiple non‐covalent interactions between PPFM and proteins, the condensates formed by this molecule can serve as transmembrane carriers, successfully enabling intracellular delivery of proteins (Figure 12C,D). Apart from BPS induced by external stimuli, the supramolecular amino acid‐encoded nanodots (SEND) developed by Duan et al. exhibited phase separation behavior that responds to ionic strength and pH conditions. (Figure 12E) [218]. Specifically, for phenylalanine‐arginine‐modified SEND (SEND‐FR), enhanced hydrogen bonding induced by bicarbonate enables the hardening of SEND‐FR condensates. (Figure 12F). Conversely, pH reduction triggers the reversion of the system to a homogeneous state, enabling the controlled release of loaded proteins. The delivery performance was further validated in living HeLa cells, where SEND outperformed a commercial PULS vector even in serum‐containing medium (Figure 12G).

**FIGURE 12:**
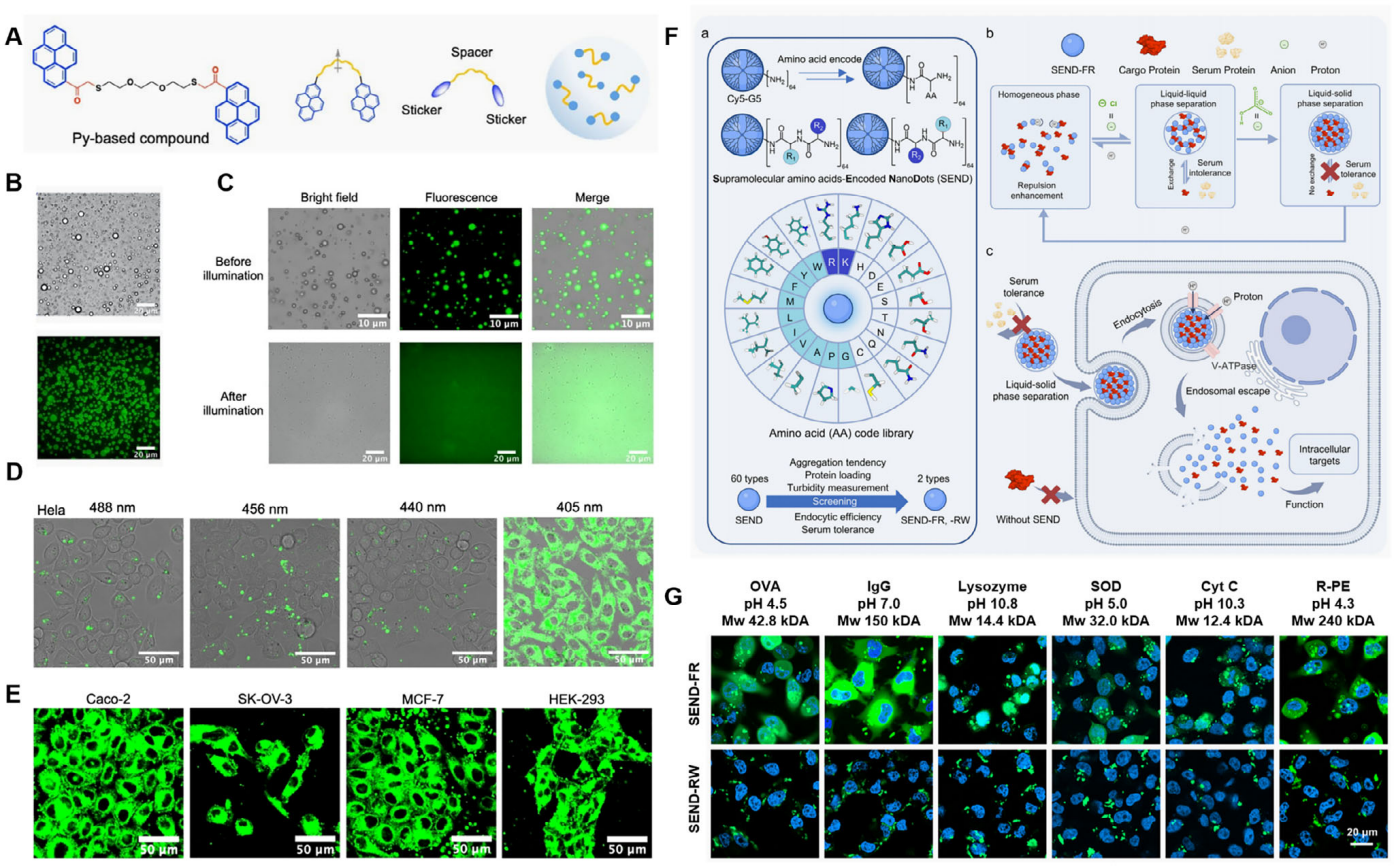
(A) Structural diagram of PPFM and schematic presentation of the stickers‐and‐spacers phase‐separation model. (B) Bright‐field and fluorescence micrographs show PPFM self‐assembles into coacervate droplets. (C) Fluorescence microscopy confirmed the disappearance of PPFM coacervates upon light irradiation. (D) Confocal imaging of HeLa cells after PPFM coacervate treatment under different light conditions. (E) Confocal observation of multiple cell lines verified that phototriggered coacervate disassembly [215]. Copyright 2023, Wiley‐VCH GmbH. (F) Schematic design and screening of phase‐separative supramolecular amino acid‐encoded nanodots (SEND) for high‐efficiency protein delivery. (G) CLSM images of HeLa cells after 4 h incubation with SEND or commercial PULS in delivery vectors under varying FBS concentrations [216]. Copyright 2025, Wiley‐VCH GmbH.

#### 5.3.2 In Situ Local Drug Enrichment

The concentration‐dependent assembly characteristic of BPS enables the condensation of biomolecules from a dispersed state to a condensed state (with a concentration hundreds of times higher than that of the dilute phase), endowing it with the potential for molecular enrichment. Similar selective partitioning behaviors of biomolecules can also be observed in all‐aqueous two‐phase systems. The regulatory principles governing molecular distribution therein offer new strategies to further optimize the bioanalytical transport functions of phase‐separated systems, such as interfacial enrichment and compartmentalized delivery [219]. It can achieve local drug enrichment at targets through active phase separation, addressing the issues of drug dispersion in non‐target tissues and insufficient concentration at target sites in traditional delivery systems [220]. This is particularly applicable to scenarios dependent on drug concentration, such as tumor therapy and transcriptional regulation. Based on this mechanism, Liang et al. adopted a bioorthogonal “click‐to‐release” strategy to in situ form biomolecular condensates as drug reservoirs. The synthesized histone‐DNA conjugate (2E‐conjugate) achieved closed‐phase separation via the click reaction between trans‐cyclooctene (TCO) and tetrazine (Tz) groups in vitro (Figure 13A) [221]. Subsequently, the release reaction in cells significantly activated LLPS. The formed biomolecular condensates could recruit the anticancer drug doxorubicin (Dox) and avoid efflux caused by overexpression of efflux pumps. The consequent drug accumulation in tumor tissues was confirmed *ex vivo* (Figure 13B,C). The clustered regularly interspaced short palindromic repeats‐associated protein 9 (CRISPR‐Cas9) system is widely used in genome engineering and transcriptional regulation, but its low transcriptional activation efficiency and complex components greatly limit its application in vivo gene therapy. Liu et al. fused the dCas9‐VPR transcriptional activation system with the IDR of phase‐separating proteins, promoting condensate formation through multivalent interactions and significantly enhancing the local concentration and activation efficiency of dCas9‐VPR [222].

**FIGURE 13:**
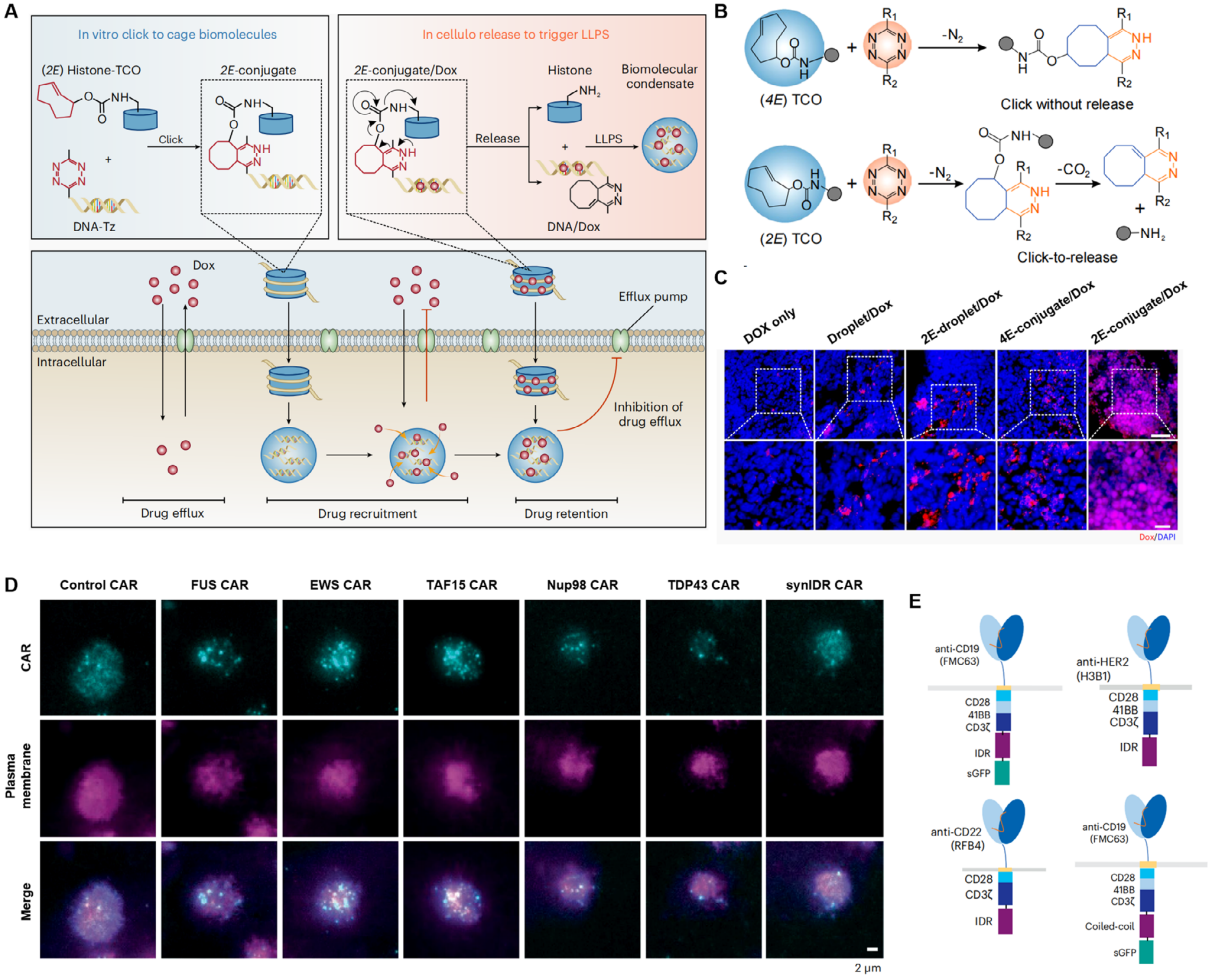
(A) Schematic design and working mechanism of an LLPS‐based intracellular drug reservoir constructed via click‐to‐release bioorthogonal reactions. Histones and DNA are separately functionalized with (2E) TCO and Tz moieties to form protein‐DNA conjugates. Caged amino groups in these conjugates temporarily suppress their phase‐separation capability. (B) Two distinct bioorthogonal reaction modes adopted in this system. The click‐to‐release reaction occurs between (2E) TCO and Tz groups, whereas the click‐to‐conjugation reaction between (4E) TCO and Tz groups proceeds without uncaging pre‐modified molecules. (C) *Ex vivo* analysis of drug accumulation in tumor tissues. Scale bars, 50 µm (top) and 20 µm (bottom) [221]. Copyright 2024, The Author(s), under exclusive licence to Springer Nature Limited. (D) TIRF microscopy reveals membrane‐localized condensation of CAR proteins. (E) Schematic representations of the engineered CD19, HER2 and CD22 CAR constructs [226]. Copyright 2025, Springer Nature.

In addition to directly regulating local drug concentrations, this strategy can significantly modulate the signal intensity of cell surface receptors. Protein drugs often induce side effects due to insufficient activation or off‐target toxicity during systemic administration. How to achieve precise regulation of the spatial distribution and aggregation of receptors by protein drugs is a core challenge for improving efficacy and safety. Ren et al. proposed an immunoglobulin‐guided phase separation system (IgPS), which utilizes the interaction between antibodies and multivalent peptides to drive the formation of condensates of specific receptors on the cell membrane, thereby enabling precise receptor aggregation and signal amplification [223]. Building on this receptor‐clustering strategy, rationally designed peptide‐based agents have been developed to trigger in situ phase separation on target cell surfaces, enabling localized drug enrichment and signaling modulation with high spatiotemporal precision [224]. For example, multivalent peptide conjugates can undergo selective coacervation with specific membrane glycans or receptors, forming stable surface condensates that prolong drug retention and enhance receptor activation potency. This cell‐surface targeting strategy has also been applied to modulate tumor cell mechanotransduction and immune recognition, offering a versatile membrane‐targeted platform for therapeutic intervention [225]. Chimeric antigen receptor T‐cell (CAR‐T) therapy has achieved unprecedented success in the treatment of hematological malignancies, but its antigen sensitivity is much lower than that of natural T‐cell receptors (TCRs) since hundreds of antigen molecules are required to activate CAR‐T cells, whereas natural T cells can be activated by a single major histocompatibility complex (MHC)‐peptide complex. This low sensitivity severely limits the efficacy of CAR‐T against tumors with low antigen expression. Zhang et al. leveraged the biomolecular phase separation property of IDRs and for the first time fused IDRs into the CAR structure through genetic engineering, inducing CAR condensation on the T‐cell surface, enhancing proximal membrane signaling, and improving antigen sensitivity (Figure 13D) [226]. CARs targeting CD19, CD22, and HER2 designed based on this strategy significantly enhanced the binding of CAR‐T cells to cancer cell targets, the mechanical strength of CAR‐T synapses, and proximal membrane signal transduction (Figure 13E).

### 5.4 Bioactive Scaffolds for Tissue Engineering

Biological scaffold materials serve a pivotal role in tissue engineering and regenerative medicine by providing a three‐dimensional framework to support cell growth, differentiation and tissue repair. Traditional biological scaffolds, such as polymer‐based scaffolds and natural scaffolds derived from natural proteins, suffer from poor biocompatibility, mismatched mechanical properties, and structural deficiencies [227]. Phase separation materials offer new opportunities to overcome these limitations. Their membrane‐less architecture mimics the porosity and spatial organization of the extracellular matrix. This structural feature enables the precise enrichment and compartmentalized release of bioactive molecules via specific intermolecular interactions, thereby constructing a favorable biomimetic microenvironment. Their dynamic responsiveness allows scaffold properties to adapt to cellular states and environmental changes, achieving multidimensional matching with cells [228]. Meanwhile, their hierarchical assembly capacity enables the reconstruction of multiscale structures ranging from nanoscale fibers to millimeter‐scale bulk scaffolds, better accommodating cell migration and tissue regeneration [229]. A full spectrum of condensate‐derived scaffold systems with diverse mechanical and biological performances has been summarized in recent comprehensive research, which reinforces the rationality of the material design logic based on phase transition types [230]. These unique features of phase separation provide a new pathway for overcoming the limitations of traditional scaffolds and designing next‐generation bioactive materials.

Inspired by the crucial role of the alternating hydrophobic and crosslinking domains of tropoelastin in driving phase separation assembly, Xie et al. proposed a biomimetic heterogeneous hydrogel system combining biomolecular phase separation and controllable covalent crosslinking (Figure 14A) [49]. By conjugating hydrophobic naphthyl (Nap) groups to hydrophilic gelatin, the phase separation propensity could be precisely regulated by adjusting the valency and interaction strength of the hydrophobic moieties, mimicking the extracellular condensation process of tropoelastin (Figure 14B). Subsequent covalent bonding of crosslinking domains triggers a condensate‐to‐hydrogel transition, forming a heterogeneous hydrogel network that stabilizes the phase‐separated structure and mimics elastin fibrosis. As a biomimetic matrix, this heterogeneous hydrogel effectively promotes mechanosensing and spreading of adherent stem cells. Gelatin hydrogels have attracted considerable interest as tissue engineering scaffolds owing to the complete degradability, intrinsic cell‐adhesive properties, and excellent biocompatibility. Conventional hydrogels constructed with the single network structure frequently exhibit inferior mechanical properties. To address this issue, Ren et al. employed a photo‐triggered transient radical and persistent bond coupling (PTPC) strategy (Figure 14C) [231]. First, radical polymerization forms dispersed hard segment particles of methacrylated gelatin (GelMA), followed by coupling between the growing radicals of GelMA and the nitrobenzyl alcohol (NB) groups of nitrobenzyl alcohol‐modified gelatin (GelNB) to construct phase‐separated structures and enhance interphase bonding (Figure 14D). The resulting h‐GelMA gel retains inherent biocompatibility while achieving significantly improved strength (10 MPa), which is compatible with 3D printing and volumetric printing technologies, offering a high‐performance scaffold material for tissue engineering and regenerative medicine (Figure 14E,F).

**FIGURE 14:**
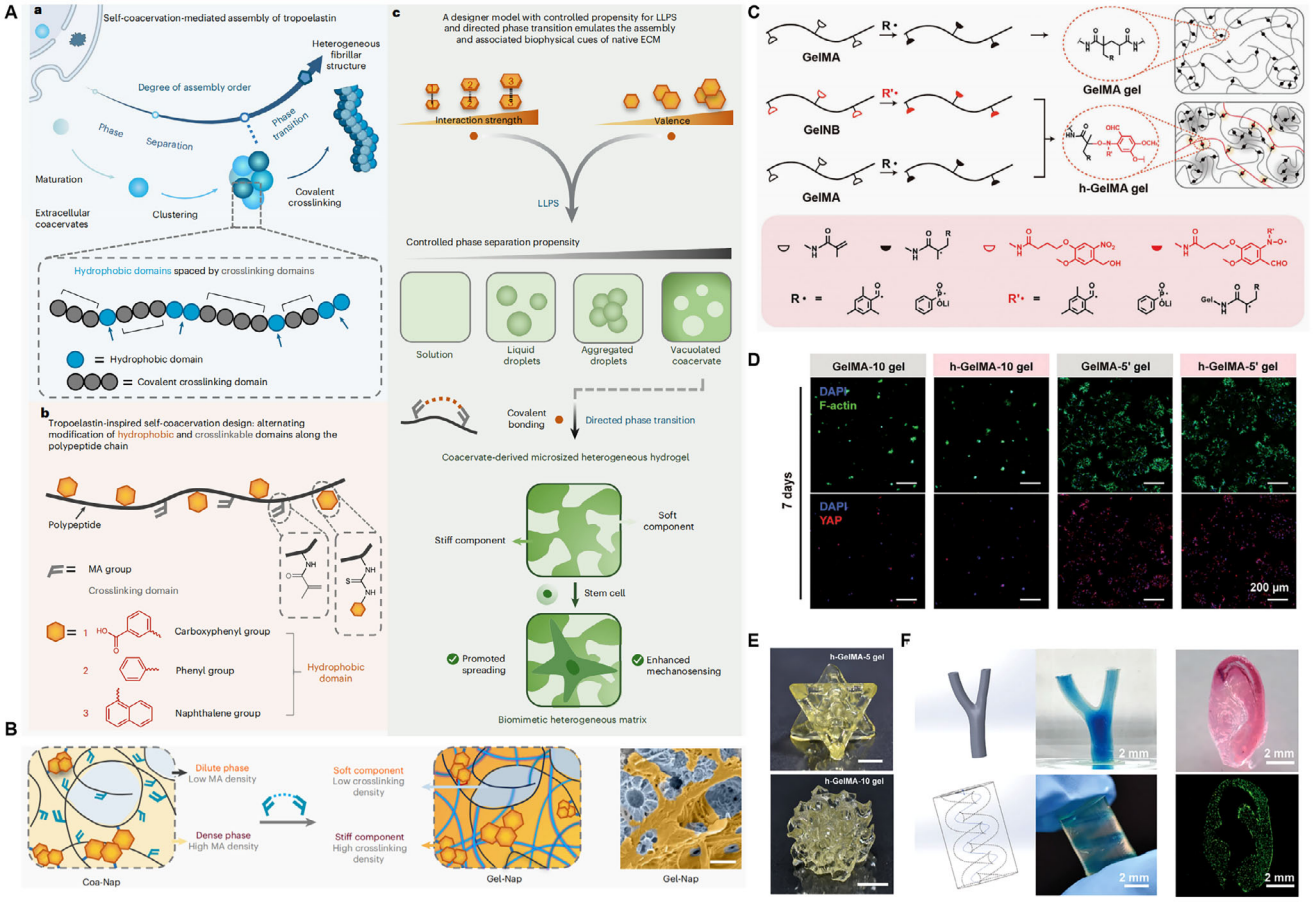
(A) A tunable minimal assembly model recapitulates the spatiotemporal organization and biophysical characteristics of the ECM to modulate cellular behavior. (a) Biomolecular coacervates act intra‐ and extracellularly to promote the ordered assembly of tropoelastin and other ECM components. (b) A tropoelastin‐mimetic minimal model constructed with alternating hydrophobic segments and covalent crosslinking domains. (c) Modulation of hydrophobic units and crosslinking‐mediated covalent bonding precisely tunes model assembly kinetics, enhances phase‐separation propensity, and recapitulates the spatiotemporal and heterogeneous properties of native ECM. (B) Schematic of phase‐separated Coa‐Nap condensates and their transformation into Gel‐Nap hydrogels [49]. Copyright 2025, The Author(s), under exclusive licence to Springer Nature Limited. (C) Comparative illustration of crosslinking mechanisms for h‐GelMA via PTPC reaction and conventional GelMA through radical polymerization. (D) Fluorescence imaging of F‐actin and YAP distribution in BMSCs encapsulated in four types of GelMA‐based hydrogels. (E) Schematic comparison of the structural stability and self‐supporting capacity of h‐GelMA‐10 and GelMA‐5 hydrogels for complex 3D biomimetic scaffold fabrication. (F) Printing prototypes and corresponding macroscopic structures fabricated using h‐GelMA‐10 hydrogel materials [231]. Copyright 2024, Wiley‐VCH GmbH.

### 5.5 Fluorescent Probes for Biomolecular Imaging

Active probes play a key role in biomedical detection and disease diagnosis by specifically recognizing and labeling biomolecules, physiological signals, or pathological microenvironments. BPS offers new insights for probe design, with features including dynamic reversibility, molecular enrichment, and inherent selectivity. Phase‐separated condensates can undergo phase transitions in response to environmental changes, enabling real‐time dynamic monitoring [232]. Their highly concentrated internal environment enhances probe‐target binding efficiency. Moreover, specific probes can be designed by modulating phase separation conditions, providing new strategies for precise detection.

Visualizing the spatial distribution and dynamics of chromosomes and extrachromosomal DNA (ecDNA) is crucial for understanding how they induce cellular dysfunction and drive disease initiation and progression [233]. However, real‐time imaging, labeling, and tracking of specific DNA sequences in living cells‐especially non‐repetitive sequences (∼65%), which constitute the majority of the genome‐remain highly challenging. Conventional fluorescence in situ hybridization (FISH) methods typically rely on cell fixation and DNA denaturation, only yielding static cellular data [234, 235]. In contrast, genomic imaging via integration of large arrays of artificial DNA sequences can perturb target loci and induce unpredictable side effects. Based on a phase separation‐regulated local signal amplification strategy, Lyu et al. developed a CRISPR‐mediated fluorescence in situ hybridization amplifier (CRISPR FISHer) system (Figure 15A) [236]. This system utilizes engineered single‐guide RNA (sgRNA) and protein trimerization domains to mediate the exponential assembly of phase separation‐based fluorescent proteins at CRISPR target sites, enhancing local brightness and signal‐to‐background ratio (SBR) to enable targeted visualization of endogenous non‐repetitive DNA loci in living cells (Figure 15B,C). Similarly, Yang et al. proposed a novel bioimaging strategy termed “condensation‐induced emission (CIE)” [237]. By covalently conjugating the aggregation‐induced emitter (AIEgen) tetraphenylethylene (TPE) with short phase‐separating peptides (PSPs), they synthesized fluorescent phase‐separating short peptide probes (Figure 15D). Upon binding to RNA, these probes drive phase separation to form stable, luminescent droplet‐like condensates (Figure 15E). Leveraging the high biocompatibility and concentration‐dependent aggregation of condensates, the phase‐separating short peptide probes achieve specific, and strong fluorescent signal imaging of intracellular RNA (Figure 15F).

**FIGURE 15:**
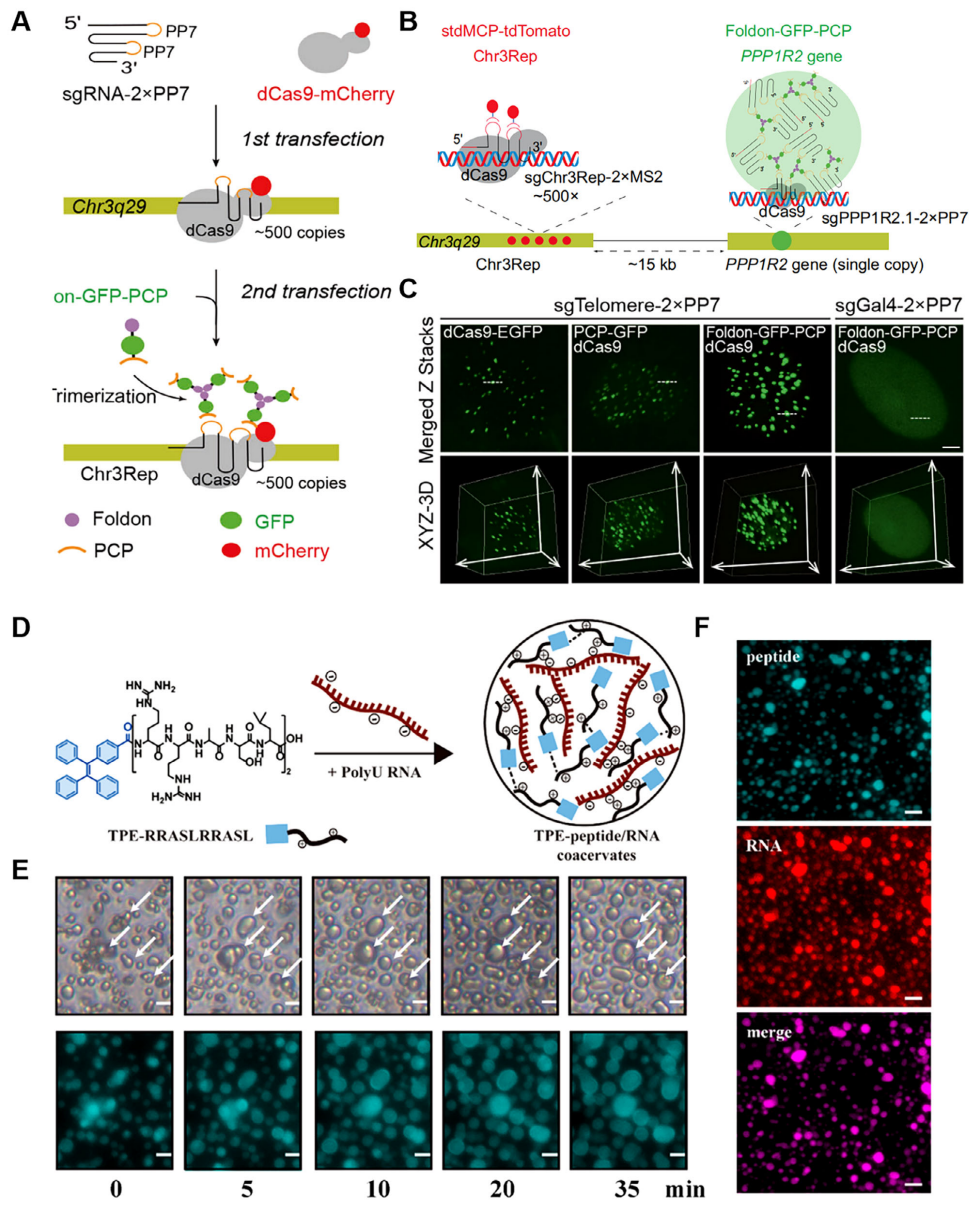
(A) Schematic recruitment mechanism of the CRISPR FISHer system to genomic target sites. (B) Schematic design of dual‐color CRISPR imaging for simultaneous visualization of PPP1R2 and Chr3Rep loci. (C) Comparative evaluation of telomere labeling performance among foldon‐GFP‐PCP, conventional PCP‐GFP and dCas9‐EGFP systems in U2OS cells. Scale bars, 10 µm [236]. Copyright 2022, Springer Nature. (D) Synthetic route of TPE‐(RRASL)n luminogens and their spontaneous coacervation with polyU RNA. (E) Time‐lapse bright‐field and fluorescence imaging records the dynamic fusion of TPE‐(RRASL)2 condensates. Scale bars, 10 µm. (F) Fluorescence imaging confirmed the colocalization of TPE‐(RRASL)n and polyU RNA within the assembled droplets. Scale bars, 10 µm [237]. Copyright.2023 American Chemical Society.

BPS has inspired four distinct classes of functional biomaterials, capitalizing on the self‐assembling and stimuli‐responsive nature of condensates. Nonetheless, current designs largely replicate phase separation phenomenology rather than systematically engineering material performance from physicochemical first principles. The long‐term in vivo stability, biodegradation profiles, and tissue compatibility of most phase‐separated materials remain undercharacterized, and scalable manufacturing with consistent quality presents substantial technical hurdles. Deeper integration of physical chemistry and biomedical engineering will enable predictable, application‐tailored material design and accelerate the translation of these proof‐of‐concept systems into clinical practice.

## 6 Challenges and Prospects

This review systematically charts the full continuum of biomolecular phase separation across liquid‐liquid, liquid‐gel and liquid‐solid states, defining their distinct physiological roles in subcellular organization, signal transduction and metabolic homeostasis. We further dissect how dysregulated phase transitions drive neurodegeneration, cancer and autoimmune disorders via four interconnected pathological cascades, and establish a property‐design‐function paradigm linking phase separation principles, targeted therapeutic strategies and BPS‐inspired functional biomaterials. This framework ties physicochemical fundamentals, disease mechanisms and translational applications into a unified cross‐disciplinary perspective.

The most fundamental barrier to clinical translation is the absence of a quantitative, programmable molecular grammar for phase state engineering. Current understanding of multivalent interactions and phase transition kinetics remains largely qualitative, forcing both drug discovery and biomaterial design to rely on empirical screening rather than rational engineering. This gap also underpins the poor selectivity of existing condensate modulators, as physiological and pathological condensates share core interaction modes. Moving forward, integrating biophysical reconstitution, AI‐driven sequence prediction and high‐throughput experimental validation will build predictive mapping between molecular features and phase behaviors, enabling selective targeting of pathological condensates and tunable design of functional materials.

Two interconnected technical bottlenecks further slow translational progress. First, the field lacks in situ characterization tools to quantitatively track condensate dynamics in native living tissues. Most mechanistic insights derive from simplified in vitro systems, leaving unresolved questions about disease causality and preventing real‐time pharmacodynamic assessment in vivo. Second, current delivery systems cannot achieve subcellular precision targeting of specific condensates, and standardized evaluation protocols for BPS‐based therapeutics and materials remain largely absent. Addressing these gaps will require multimodal imaging platforms for in vivo condensate profiling, organelle‐level targeted delivery systems, and unified preclinical evaluation pipelines to ensure result reproducibility and scalable manufacturing.

Taken together, biomolecular phase separation research is undergoing a paradigm shift from descriptive cell biology to quantitative, engineering‐driven science. As a cross‐disciplinary hub connecting physical chemistry, molecular medicine and biomedical engineering, BPS has already reshaped our understanding of cellular organization and disease pathogenesis, and holds substantial potential for treating previously intractable conditions and developing life‐like intelligent biomaterials. While multiple challenges remain, sustained cross‐disciplinary collaboration will unlock the full translational value of phase separation principles, establishing BPS as a versatile platform for precision medicine and opening new dimensions for disease intervention and tissue regeneration.

## 7 Conclusions

BPS constitutes a fundamental principle of intracellular spatial organization, operating across a dynamic continuum from liquid droplets to gel‐like assemblies and solid aggregates. This review delineates how graded phase transitions tune core physiological processes including membraneless organelle biogenesis, signal transduction, and metabolic homeostasis. Dysregulated phase state maturation drives interconnected pathological cascades across neurodegeneration, cancer, and autoimmune disorders. It transforms functional condensates into cytotoxic aggregates or aberrant signaling hubs. Building on these mechanistic foundations, the property‑design‑function paradigm enables the rational development of phase separation‑targeted therapeutics and bioinspired materials. These materials cover drug delivery platforms, tissue engineering scaffolds, as well as imaging probes. Substantial challenges regarding target selectivity and in vivo characterization still persist. Cross‑disciplinary integration of biophysics, molecular medicine, and materials science will unlock the full translational potential of phase separation principles. It will also construct a versatile framework for next‑generation precision medicine.

## Author Contributions

**Jiawei Zhu**: conceptualization, funding acquisition, writing – original draft, writing – review and editing, data curation. **Jingbo Dong**: writing – review and editing, data curation. **Qiufang Gong**: writing – review and editing, data curation. **Xuejiao Song**: conceptualization, writing – review and editing, supervision. **Zhifa Shen**: conceptualization, supervision. **Chao Liang**: resources, funding acquisition, conceptualization.

## Conflicts of Interest

The authors declare no conflicts of interest.

## Data Availability

The authors have nothing to report.
